# RegioSQM20: improved prediction of the regioselectivity of electrophilic aromatic substitutions

**DOI:** 10.1186/s13321-021-00490-7

**Published:** 2021-02-12

**Authors:** Nicolai Ree, Andreas H. Göller, Jan H. Jensen

**Affiliations:** 1 Department of Chemistry, University of Copenhagen, Universitetsparken 5, 2100 Copenhagen, Denmark; 2 Bayer AG, Pharmaceuticals, R&D, Computational Molecular Design, 42096 Wuppertal, Germany

## Abstract

We present RegioSQM20, a new version of RegioSQM (Chem Sci 9:660, 2018), which predicts the regioselectivities of electrophilic aromatic substitution (EAS) reactions from the calculation of proton affinities. The following improvements have been made: The open source semiempirical tight binding program xtb is used instead of the closed source MOPAC program. Any low energy tautomeric forms of the input molecule are identified and regioselectivity predictions are made for each form. Finally, RegioSQM20 offers a qualitative prediction of the reactivity of each tautomer (low, medium, or high) based on the reaction center with the highest proton affinity. The inclusion of tautomers increases the success rate from 90.7 to 92.7%. RegioSQM20 is compared to two machine learning based models: one developed by Struble et al. (React Chem Eng 5:896, 2020) specifically for regioselectivity predictions of EAS reactions (WLN) and a more generally applicable reactivity predictor (IBM RXN) developed by Schwaller et al. (ACS Cent Sci 5:1572, 2019). RegioSQM20 and WLN offers roughly the same success rates for the entire data sets (without considering tautomers), while WLN is many orders of magnitude faster. The accuracy of the more general IBM RXN approach is somewhat lower: 76.3–85.0%, depending on the data set. The code is freely available under the MIT open source license and will be made available as a webservice (regiosqm.org) in the near future. 
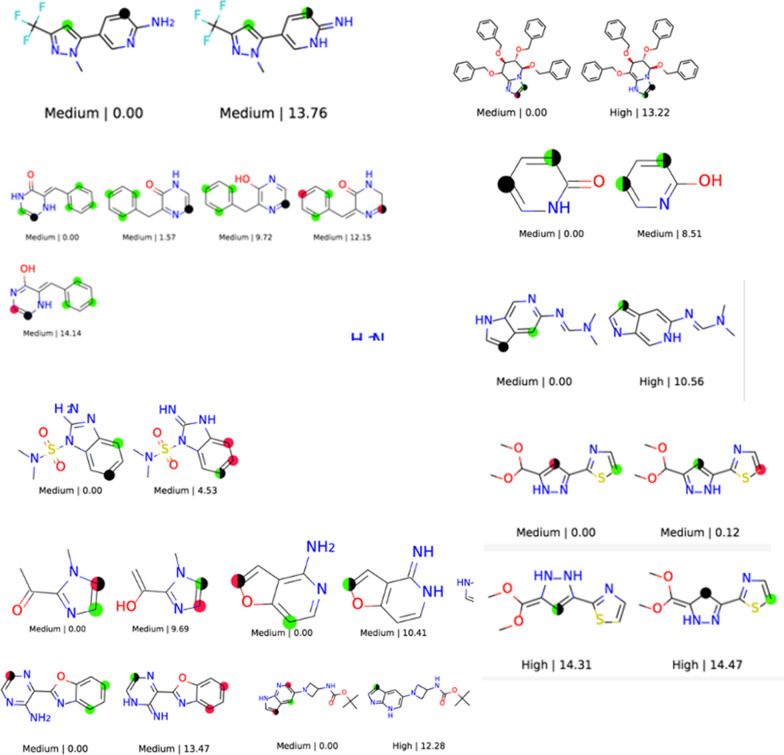

## Introduction

Halogenated derivatives of heteroaromatics and benzene derivatives are often applied as substrates in carbon-carbon and carbon-heteroatom cross-coupling reactions[1, 2] and are typically prepared by electrophilic aromatic substitution (EAS). However, it is often not a priori obvious at which position(s) halogenation will occur for compounds in the late stages of the synthesis that contain multiple (hetero)aromatic rings or in compounds that contain both heteroarene and benzene rings. Consequently, organic chemists tend to install the halogens early in the synthesis, thereby effectively eliminating a large number of otherwise promising synthetic routes. Furthermore, EAS is also an important tool in late stage functionalization [3], which utilizes the C–H bonds of drug leads as points of diversification for generating new analogs, if the regioselectivity can be predicted.

Several predictive tools have been developed to address this problem based on heuristics [4], quantum chemical calculations (QM),[5] machine learning (ML) [6, 7] or a combination of QM and ML [8, 9]. Furthermore, ML-based software that predict retrosynthetic pathways [10–17] are also implicitly trained to predict the regioselectivity of EAS reactions [14]. However, these methods are trained on a much broader dataset and their a gain in generality could lead to a loss in single reaction type accuracy. One of the former methods is the RegioSQM method developed by Kromann et al. [5] (referred to hereafter as RegioSQM18). RegioSQM18 uses the semiempirical PM3 method [18] and the COSMO continuum solvation model [19] implemented in the MOPAC program. MOPAC is a closed-source software package that is free to academics but not to industry, so we decided to investigate open source alternatives for further development of RegioSQM. In this paper, we show that the open source semiempirical software package xtb can be used in place of MOPAC without impacting the accuracy of the predictions. We go on to show that the accuracy can be increased by considering different tautomeric forms of the molecule and offer a qualitative prediction of the reactivity of each tautomer. Finally, we compare the accuracy of the new version (RegioSQM20) to two ML-based models for regioselectivity predictions.

## Computational methodology

**Fig. 1:**
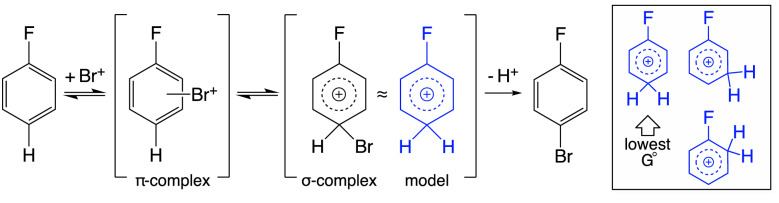
The mechanism of an electrophilic aromatic substitution (EAS) reaction using fluorobenzene as an example. RegioSQM approximates the Br $\sigma$-complex as protonation (shown as blue structures) and determines regioselectivity by finding the protonated isomer with lowest free energy

Figure 1 illustrates the EAS mechanism using the bromination of fluorobenzene as an example. The mechanism is relatively simple and involves the addition of an electrophile to the aromatic ring to form a $\sigma$-complex (also called a Wheland intermediate or an arenium ion), which usually determines the regioselectivity of EAS reactions with Br and Cl. Hence, free energy calculations of different protonated regioisomers, corresponding to different $\sigma$-complexes and thereby different reaction pathways, can be used to predict the regioselectivity.

**Fig. 2:**
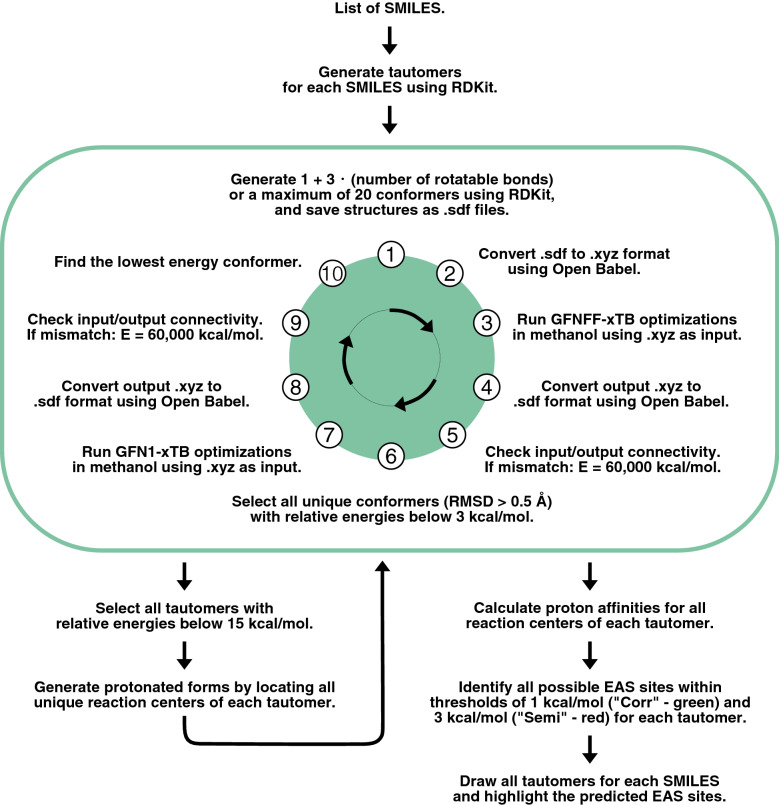
A flowchart describing the procedure of RegioSQM20

**Fig. 3:**
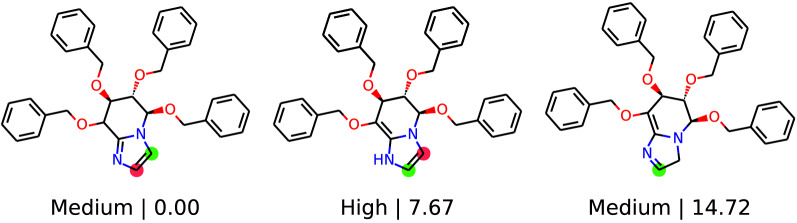
An example of the output of RegioSQM20. Tautomers are depicted with relative energies (only including those below 15 kcal/mol) along with an estimate of their reactivity based on the highest proton affinity. The highlighted atoms represent the predicted EAS sites within 1 kcal/mol (green circles) and the EAS sites within 3 kcal/mol (red circles)

The procedure of RegioSQM20 is fully automated with the only user input being a SMILES (simplified molecular input line entry system) representation of a given molecule (see Fig. 2). RegioSQM20 will then generate tautomers using TautomerEnumerator in RDKit 2020.03.1 [20] and all tautomers will go into a conformational search algorithm. Note that if a tautomer is generated adjacent to a chiral center, the output structure/SMILES will have that center removed. In this algorithm, $\text {min}(1+3\cdot n_{\text {rot}},20)$ conformers are generated for each tautomer, where $n_{\text {rot}}$ is the number of rotatable bonds. The conformers are then optimized in methanol (MeOH, dielectric = 33.6) using the fast force-field version of xTB called GFNFF-xTB and the generalized Born (GB) model with solvent accessible surface area (SASA) termed GBSA [21]. Compared to RegioSQM18, this new implementation generates conformers using EmbedMultipleConfs from RDKit with ETversion=2 instead of ETversion=1. After this procedure, all conformers with relative total energies below 3 kcal/mol are clustered with the Butina algorithm in RDKit to find unique conformers using the pairwise heavy-atom position root mean square deviation (RMSD) with a threshold of 0.5 Å. The cluster centroids are then re-optimized in MeOH using GFN1-xTB and the GBSA solvation model in order to find the lowest energy conformers [22]. After both optimizations, the input and output structures are compared by converting the Cartesian coordinate file (.xyz) into a structure-data file (.sdf) using Open Babel 2.4.1 [23]. If the atom connectivity is different, due to e.g. an intramolecular proton transfer reaction or a broken/created bond, the energy of the molecule is set to 60,000 kcal/mol. In case the force-field calculation fails, the initial RDKit structure will be used as the input structure for the GFN1-xTB calculation. Hereafter, RegioSQM20 selects all tautomers with relative total energies below 15 kcal/mol and locates all unique reaction centers to generate single protonated forms of the tautomers. These protonated molecules are then sent into the conformational search algorithm to find their lowest energy conformer. Subsequently, the proton affinities are calculated as the energy difference between the unprotonated and protonated forms. Note that we neglect the energy of the proton in solution, since the qualitative reactivity categorization is based on comparing the proton affinities to cutoff values where this term is also neglected. The predicted EAS sites are then identified as the reaction centers with proton affinities within 1 kcal/mol (“Corr”-green) and 3 kcal/mol (“Semi”-red) of the highest proton affinity. Finally, the tautomers are drawn and their respective EAS sites are highlighted as seen in Fig. 3. All tautomers are labelled with the relative energies of the unprotonated forms and the predicted reactivity based on the highest proton affinity of each tautomer.

The CPU timings are obtained on four Intel(R) Xeon(R) CPU X5550 @ 2.67GHz cores. The source code is freely available on GitHub (https://github.com/jensengroup/RegioSQM20) and will be made available as a web service at regiosqm.org in the near future.

## Results and discussion

### Switching from PM3 to GFN-xTB

**Fig. 4:**
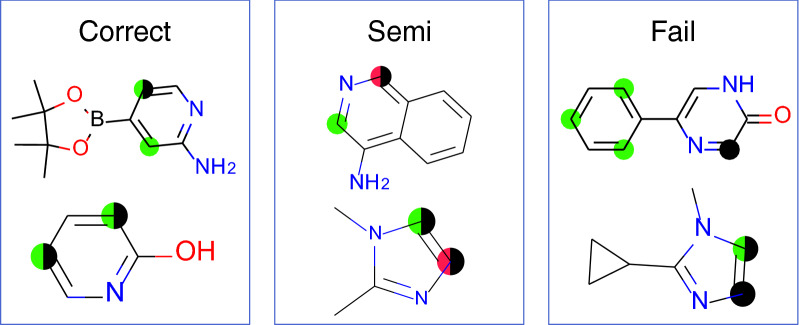
Definition of correct, semi-correct, and incorrect predictions of regioselectivity. RegioSQM correlates proton affinity with EAS reactivity. The green and red dots indicate the atoms with proton affinities within 1 and 3 kcal/mol of the highest value, respectively. The black dots indicate the experimentally observed sites for EAS. All experimentally observed sites must be predicted by green dots to be counted as correct

RegioSQM18 was developed and tested using 535 EAS reactions collected from the literature [5] and we use the same data set to guide the development of RegioSQM20. The dataset includes twenty monocyclic systems ranging from pyrrole to 1,2,4-triazine-3,5(2H,4H)-dione and 64 bicyclic systems. Important aromatic systems like benzene and pyridine as well as indazole and 7-azaindole are well-represented with 16-214 examples, but the analysis also includes a number of less common heteroaromatics like pyridazin-3(2H)-one and imidazo[1,2-a]pyrimidine with 1 and 2 examples, respectively. See reference [5] for more information. RegioSQM18 predicts the correct regioselectivity for 488 of the 535 reactions, while 30 and 17 are predicted semi-correctly and incorrectly, respectively (Table 1). A correct prediction is one where all experimentally observed sites have predicted proton affinities within 1 kcal/mol of the highest proton affinity (marked as green in Fig. 4). The definition of a semi-correct prediction is the same as a correct prediction except that the 1 kcal/mol cutoff is changed to 3 kcal/mol (marked as red in Fig. 4). Finally, an incorrect (or failed) prediction is one where at least one experimentally observed site is not predicted correctly. We repeated the calculations with GFN1-xTB and GFN2-xTB in combinations with a variety of solvents and found that GFN1-xTB and methanol gave the most accurate results with 486, 27, and 22 correct, semi-correct, and failed predictions (Table 1 and Additional file 1: Fig. S1). This result is very similar to those obtained with PM3 and shows that an open source method can be used instead of PM3.

**Table 1:** Comparing RegioSQM implementations

Methodology	Corr/Semi/Fail	Median CPU time (s)$^{\text{a}}$	Mean CPU time (s)$^{\text{a}}$	Total CPU time (h)$^{\text{a,b}}$
RegioSQM18	488/30/17	42	127	10
GFN1-xTB/methanol	486/27/22	60	230	17
FF Optimization	485/29/21	33	110	8
Tautomers (canonical RDKit)	477/28/30	33	110	8
Tautomers (lowest energy)	483/27/25	39	163	12
Tautomers (15 kcal/mol)	496/21/18	49	223	17

The last entry corresponds to RegioSQM20. For the generation of the conformers, a random seed of 90 was used. Furthermore, the RegioSQM18 and GFN1-xTB/methanol entries uses ETversion=1 and otherwise ETversion=2. Corr/Semi/Fail is defined in Fig. 4

$^{\text{a}}$4 cores/molecule (Intel(R) Xeon(R) CPU X5550 @ 2.67GHz). $^{\text{b}}$Two molecules running in parallel

Using the GFN1-xTB/GBSA method increases the median CPU time requirement from 43 to 60 s and the total time for all 535 increases from 10 to 17 h. In order to mitigate the increased computational cost, we tested the use of the GFNFF-xTB force field to pre-screen the conformers. If only conformers with GFNFF-xTB/methanol energies within 3 kcal/mol of the lowest energy conformer are re-optimized using GFN1-xTB/methanol then the number of correct, semi-correct, and failed predictions (485, 29, and 21) are essentially unchanged (Table 1) while decreasing the median CPU time per molecule to 33 s and total time to 8 h.

### Tautomers

The experimental regioselectivity data we use is collected from the literature and the tautomeric forms of the molecules in that data set are those displayed in the respective publications. A few exploratory calculations revealed that the tautomeric form of the molecule can have an impact on the predicted regioselectivity, so we decided to address this issue in a more systematic fashion. If we instead use the tautomer with the lowest GFN1-xTB/methanol energy the number of correct predictions is 483 (Table 1), which is slightly lower than that obtained without considering tautomers. The most probable explanation is that GFN1-xTB/methanol is not sufficiently accurate to identify the most stable tautomer and/or that this property is more sensitive to the choice of solvent. We therefore investigated the effect of including all tautomers within a certain cutoff of the lowest energy form and the results are shown in Additional file 1: Fig. S2. The figure shows that a relatively large cutoff (> 8 kcal/mol) is needed in order for the inclusion of tautomers to have a significant effect on the accuracy.

A correct prediction is one where all the experimentally observed sites are predicted (with a green dot) by at least one of the tautomers, so one worry is that the high cutoff simply leads to a large number of tautomers each with a different site being predicted as most reactive. However, an analysis of the data (Additional file 1: Fig. S3) shows that 91% of the molecules in our data set have only one or two tautomers, even for a cutoff of 15 kcal/mol. This statistic is reflected in the 12 molecules for which the predictions improve using a cutoff of 15 kcal/mol (Additional file 1: Fig. S4). Of these 12 molecules only three have more than two tautomers and in all but one case the increase in accuracy is a result of only one new position being predicted as most reactive (Additional file 1: Fig. S4). Thus, RegioSQM20 uses a 15 kcal/mol cutoff for tautomers, which increases the number of correct predictions to 496 molecules (Table 1) and the median and total CPU time to 49 s and 17 h, respectively.

The CPU time can be significantly reduced by using GFN2-xTB with only a relatively minor decrease in accuracy. For example, GFN2-xTB/water has a median CPU time of only 29 s and total time of 10 h, while the number of correct and incorrect predictions are 493 and 24, respectively. Thus, this method can be selected if computational efficiency is a greater concern than accuracy.

### Comparison to machine learning models

Several machine learning (ML) based models predict regioselectivity of EAS reactions and have been compared to RegioSQM18, such as the Weisfeiler-Lehman neural network (WLN) based method by Struble et al. [6] and the molecular transformer (MolTrans) by Schwaller et al. [14].

Struble et al. compared the top one, two, and three predictions of the WLN-based method, which predicts a reaction probability for each atom, to those of RegioSQM18 and found success rates of 85.0–95.7% for WLN, compared to 79.7–93.3% for RegioSQM18. However, this approach does not consider the differences in the reaction probabilities (in the case of WLN) nor relative energies of the protonated isomers (in the case of RegioSQM). For example, a top-2 prediction would be correct even if the second reaction probability is extremely low, like for instance 98% and 6% for the first and second position, and similarly for protonated isomers with relative energies of 0 and 15 kcal/mol for RegioSQM. Conversely, a top-1 prediction would be considered incorrect even if the top two reaction probabilities are 99% and 98%, and the reaction is observed to occur at the site with a 98% reaction probability. Or, in the case of RegioSQM, if the reaction is observed to occur at a site where the corresponding isomer is 0.2 kcal/mol higher than the isomer with the lowest energy. Instead, we therefore define a correct WLN prediction if the observed reaction site has a reactivity score that is within 15% of the highest score, and a semi-correct otherwise as long as the prediction probability is >5% (the cutoff the authors used to classify non-reactive atoms). With these definitions, and without considering tautomers, the number of correct and incorrect WLN-based predictions are 477 and 32, respectively, compared to 485 and 21 for RegioSQM20* (“FF optimization” entry in Table 1, i.e. without considering tautomers). This corresponds to a success rate of 89.1% and 90.7% for WLN and RegioSQM20*, respectively, while the corresponding failure rates are 6.0% and 3.9% (Table 2). For comparison the success and failure rates for RegioSQM20 with tautomers are 92.7% and 3.4%, respectively.

**Table 2:** Comparison of RegioSQM to two ML-based models. Tautomers are not considered so “RegioSQM20*” (note the “*”) corresponds to the “FF Optimization” entry in Table 1

Methodology	Full data set	One reactive atom
	Corr/Semi/Fail	Corr/Fail
RegioSQM20*	90.7%/5.4%/3.9%	92.0%/8.0%
WLN	89.1%/4.9%/6.0%	96.0%/4.0%
IBM RXN	76.3%/23.7%	85.0%/15.0%

“One reactive atom” refers to the subset of the full data set with only one unique experimentally observed reaction site and where RegioSQM20* only predicts one unique (green) reactive site

Since the chosen cutoffs are somewhat arbitrary, we also investigate the subset of 426 molecules with only one unique experimentally observed reaction site and where RegioSQM20* only predicts one unique (green) reactive site. Here, RegioSQM20* is compared to the top-1 WLN prediction and the success rates for WLN and RegioSQM20* are 96.0% and 92.0%, respectively. A similar comparison to RegioSQM18 by Struble et al. for a different set of molecules (not used to train the WLN method) yielded 87.9% and 86.7%, respectively. The relatively large difference in success rates observed for WLN for these two sets of molecules could indicate that many of the molecules used in the current data set are included in the WLN-training set. Overall, the success rates of RegioSQM20 and the WLN-based method are thus comparable, while the latter is orders of magnitude faster.

The development of the techniques underlying MolTrans has been continued in the IBM RXN for Chemistry (IBM RXN) package [15] so this is the package we compare to RegioSQM20. We use N-bromosuccinimide (NBS) as the reagent since this is the source of Br for most of the reactions in our data set and, as before, we use the tautomeric form found in the data set. With IBM RXN we only have access to the most likely prediction and with this limitation the success rate is 76.3% using the entire data set. This is somewhat lower than the top-1 success rate of 83% reported for MolTrans by Schwaller et al. for a different data set. It is not clear whether the difference is due to differences in the data set or differences between MolTrans and IBM RXN. However, the corresponding success rate for the 426 molecules with only one unique experimentally observed reaction site and where RegioSQM20* only predicts one unique (green) reactive site is 85.0%, which is closer to the value reported by Schwaller et al. and somewhat lower than the 96.0% and 92.0% observed for WLN and RegioSQM20*.

### Prediction of reactivity

**Fig. 5:**
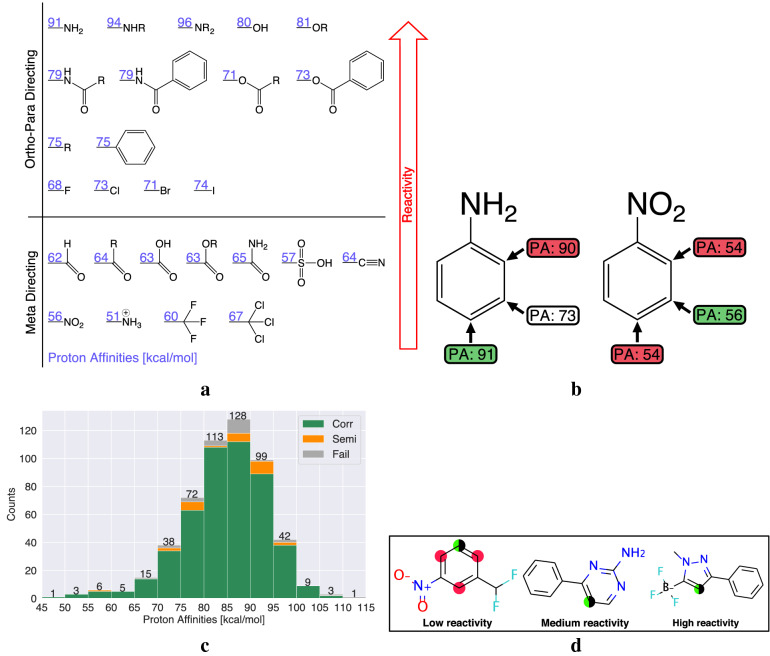
**a** The highest proton affinity of the most stable tautomer computed for a series of substituted benzene analogs familiar to all organic chemists together with a qualitative ranking of their reactivities. The values are given in units of kcal/mol. **b** Two examples from **a** showing the proton affinities of the unique reaction centers. **c** Computed proton affinities for the 535 molecules in our data set as the energy difference between the unprotonated and most stable protonated form using the most stable tautomer. **d** Examples of molecules with predicted low, medium, and high reactivity based on the highest proton affinities

The original inspiration for using proton affinities to predict regioselectivity came from the observation by Streitwieser and others that the rates of many EAS reactions correlate well with the proton affinity of the reacting carbon [24]. While RegioSQM18 only predicts the relative proton affinities (i.e. the relative energies of the protonated isomers), the proton affinity (i.e. the energy difference between the unprotonated and protonated forms) can be calculated at no additional cost since the energy of the unprotonated form of the molecule is computed to identify low energy tautomers. The proton affinities are more difficult to obtain accurately than relative proton affinities, so we only expect these values to give a qualitative indication of reactivity. Figure 5a shows the highest proton affinity of the most stable tautomer computed for a series of substituted benzene analogs familiar to all organic chemists together with a qualitative ranking of their reactivities. There is a clear separation in the proton affinities of the most (80–91 kcal/mol) and least (51–67 kcal/mol) reactive molecules. An example from each of these classes along with the proton affinities of all the unique reaction centers can be seen in Fig. 5b.

Having established a qualitative correlation between reactivity and proton affinity computed by GFN1-xTB/methanol, we computed the highest proton affinity of the most stable tautomer for the 535 molecules in our data set. The results (5c) show that 92% of the molecules have proton affinities in the range 70–100 kcal/mol— a range similar to that found for most of the reactive (ortho-para directing) molecules shown in 5a. Molecules with higher and lower proton affinities are thus deemed unusually high and low reactivity, respectively. RegioSQM20 therefore uses these cutoffs to classify a molecule as having low, medium, or high reactivity and displays this information in the output (Fig. 5d).

## Conclusions and outlook

We present RegioSQM20, a new version of RegioSQM [5], which predicts the regioselectivities of electrophilic aromatic substitution (EAS) reactions by finding the atomic center with the highest proton affinity. The following improvements have been made: The open source semiempirical tight binding program xtb is used instead of the closed source MOPAC program; specifically GFN1-xTB/methanol is used instead of PM3/chloroform. Any low energy tautomeric forms of the input molecule are identified and regioselectivity predictions are made for each tautomeric form. The increase in CPU time associated with this capability is offset by pre-screening low energy conformations with the GNFF-xTB force field without significant loss of accuracy. The median CPU time requirements of RegioSQM20 is 49 s per molecule on four Intel(R) Xeon(R) CPU X5550 @ 2.67GHz cores, but the computational cost depends heavily on the number of possible reaction sites and number of low energy tautomeric forms. Finally, RegioSQM20 offers a qualitative prediction of the reactivity of each tautomer (low, medium, or high) based on the highest proton affinity, i.e. the energy difference between the unprotonated and most stable protonated form using the most stable tautomer.

RegioSQM20 is developed and tested on 535 molecules for which the regioselectivity of bromination by EAS has been reported in the literature. The accuracy of the predictions with xTB and PM3 are roughly the same (ca. 91%), indicating that the same accuracy can be achieved with an open source approach. The inclusion of tautomers increases the success rate from 90.7% to 92.7%.

RegioSQM20 is compared to two machine learning based models: one developed by Struble et al. [6] specifically for regioselectivity predictions of EAS reactions (WLN) and a more generally applicable reactivity predictor developed by Schwaller et al. [14]. (IBM RXN). RegioSQM20 and WLN offers roughly the same success rates for the entire data sets (without considering tautomers), while WLN is many orders of magnitude faster. The accuracy of the more general IBM RXN approach is somewhat lower: 76.3%-85.0%, depending on the data set.

While the WLN based method is considerably faster, the RegioSQM approach may perform better for ring systems that are not well represented in the training set. Unfortunately, the training sets used to develop the WLN based method is not publicly available, so this hypothesis is difficult to check. Another difference is that RegioSQM finds two or more atoms with roughly equal reactivity in about one fourth of the molecules while this almost never happens with the WLN based method. This could reflect the possibility that the literature and especially patents tend to report only the desired product even if other products are observed. This “bias” is then introduced to machine learning models since they are developed based on data that are extracted from these sources. For example, 21 out of the 32 molecules that fail with WLN (without considering tautomers) has more than one experimentally observed reactive site, compared to 6 out of 21 for RegioSQM20*. In fact, out of the 38 molecules in our data set with two or more experimentally observed reaction sites, WLN makes correct prediction for only two molecules, while RegioSQM20* makes correct predictions for 25. RegioSQM20 thus could offer a useful complement to machine learning based methods in some cases.

## Supplementary Information

**Additional file 1.** Additional Figures and Table.

## Data Availability

The code is available at https://github.com/jensengroup/RegioSQM20 and data is available at https://github.com/jensengroup/SI_RegioSQM20.
