# Supplementary material for: RegioSQM20: improved prediction of the regioselectivity of electrophilic aromatic substitutions
Source: J Cheminform. 2021 Feb 12;13:10. doi: 10.1186/s13321-021-00490-7 (PMC7881568; doi:10.1186/s13321-021-00490-7)
Supplement: Supplementary file 1 — Additional file 1. Additional Figures and Table. [file 13321_2021_490_MOESM1_ESM.pdf]

# Supporting Information

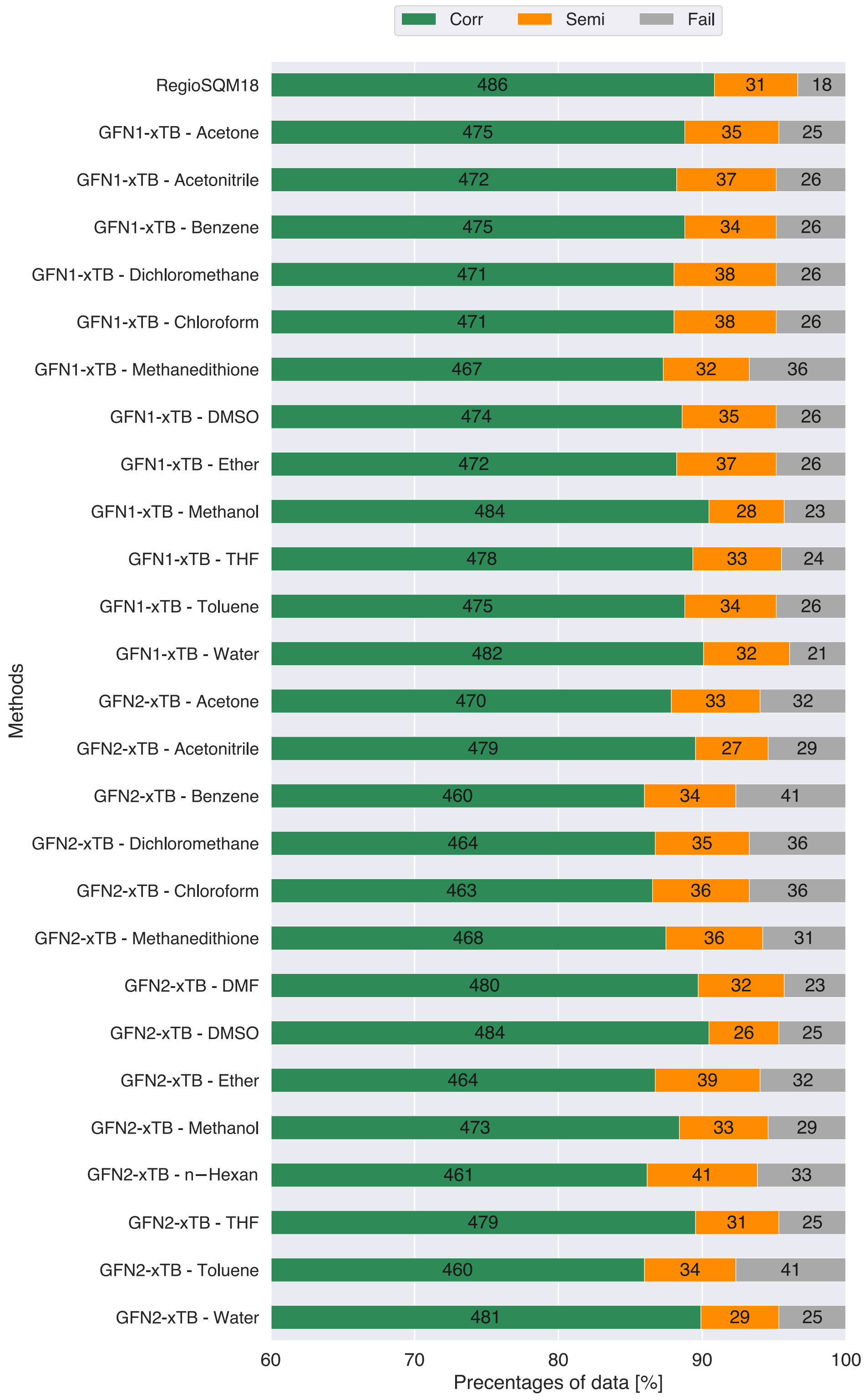


Figure S1: The results of employing different xTB methods in combination with a variety of solvents. All of the starting conformers were generated by RDKit in RegioSQM18 without a random seed, however they were exactly the same for all of the methods shown.

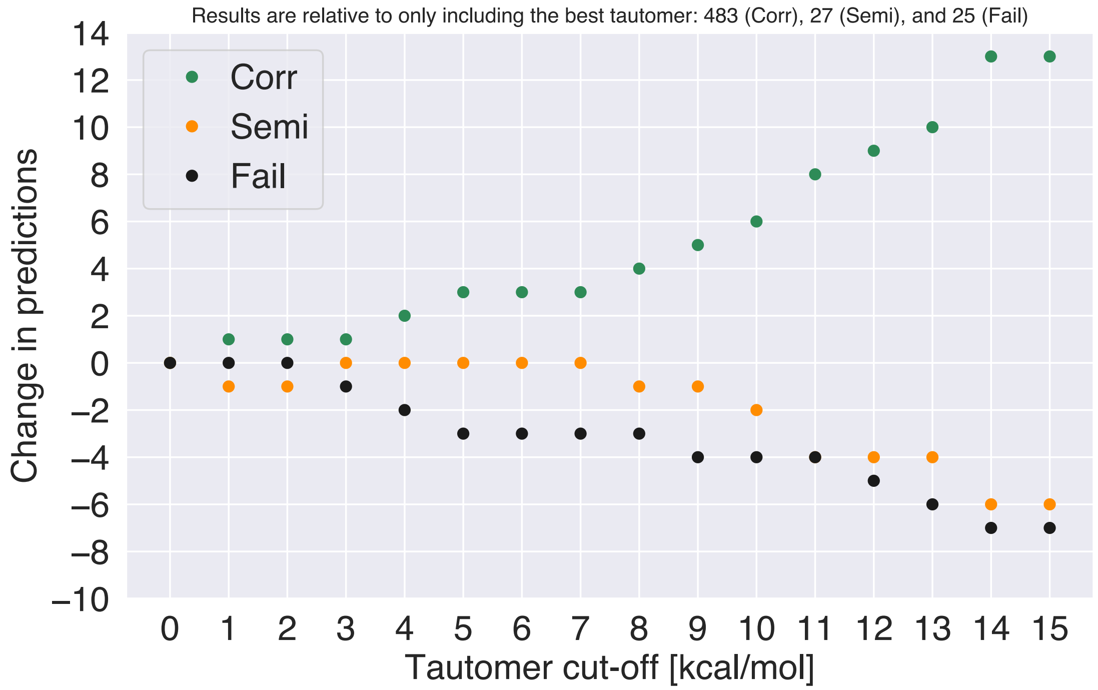


Figure S2: Change in accuracy with increased tautomer cutoff

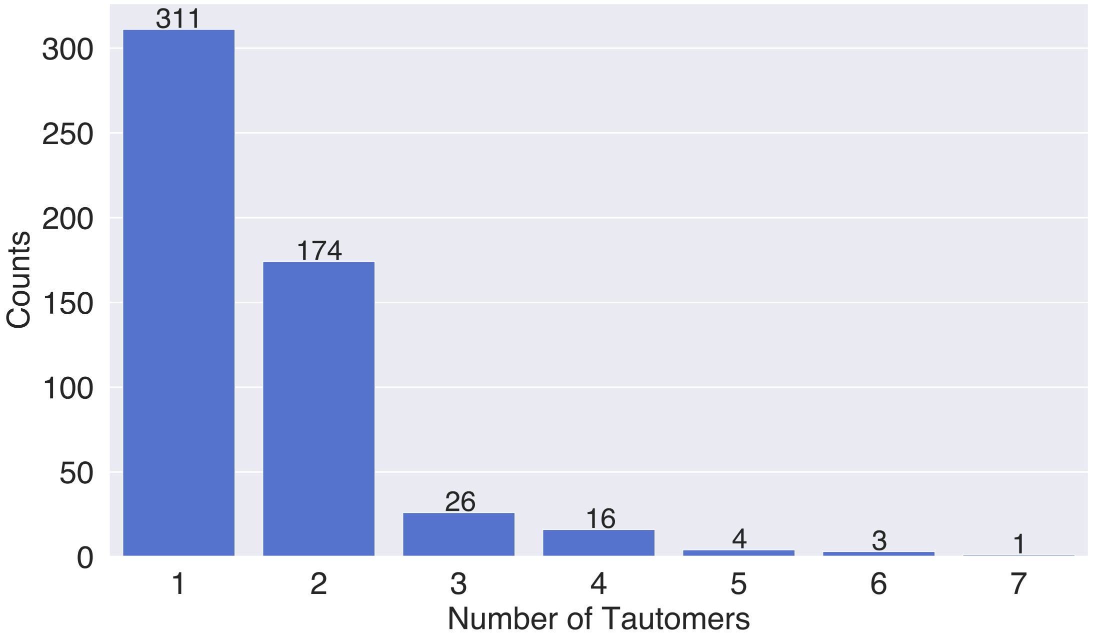


Figure S3: Histogram showing the number of tautomers for each molecule by employing RegioSQM20, which has a tautomer cut-off of 15 kcal/mol.

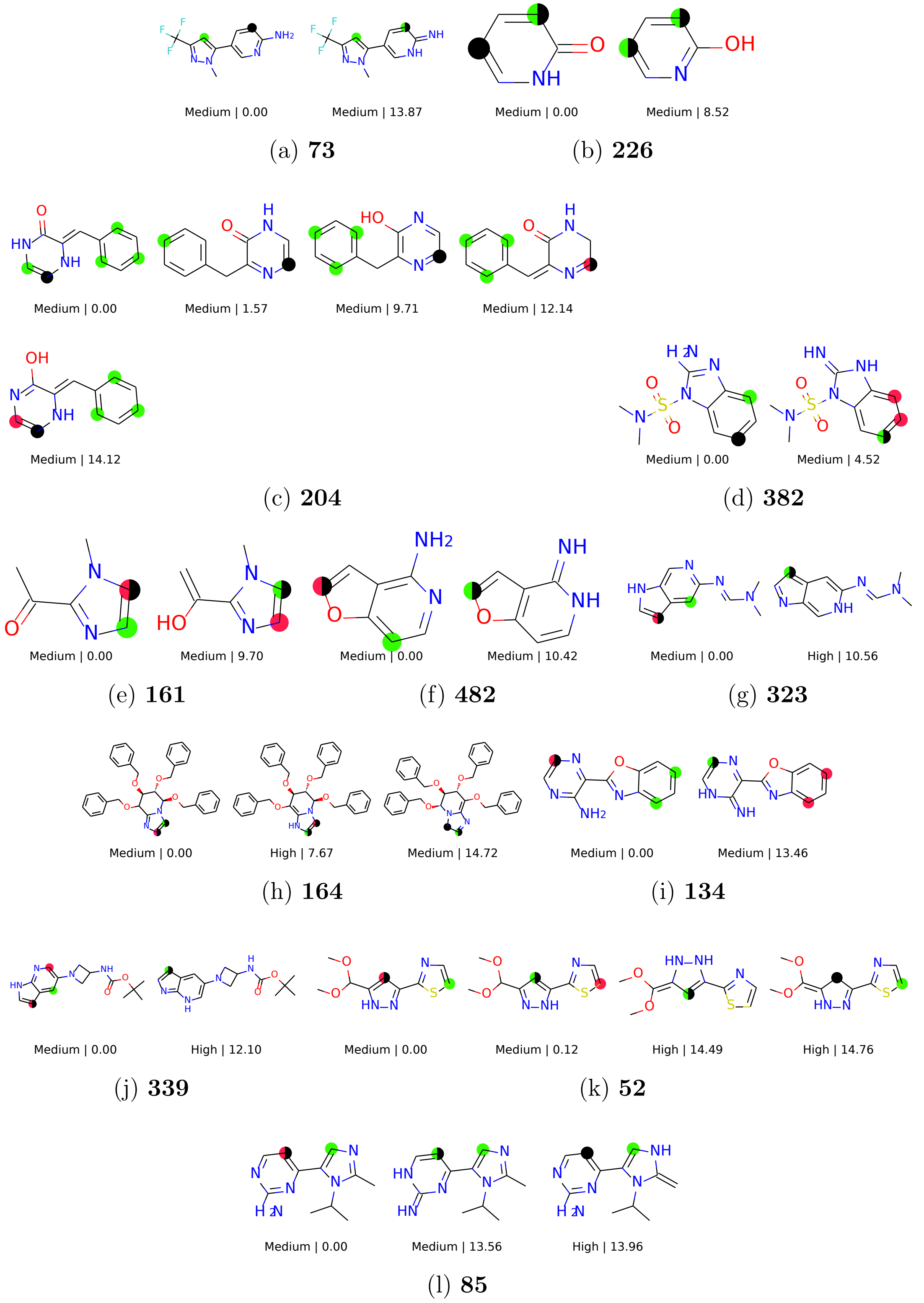


Figure S4: Molecules for which the inclusion of tautomers improves the accuracy.

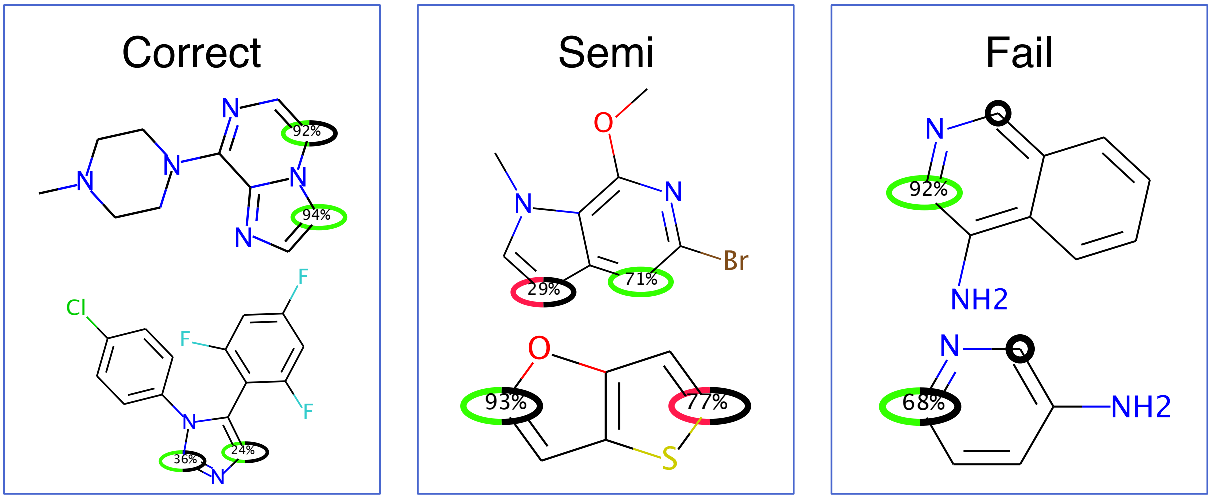


Figure S5: Definition of correct, semi-correct, and incorrect predictions of WLN. The green and red circles indicate the atoms with reactivity scores within 15% of the highest score and above 5% for atoms not within 15% of the highest score, respectively. The black circles indicate the experimentally observed sites for EAS. All experimentally observed sites must be predicted by green circles to be counted as correct.

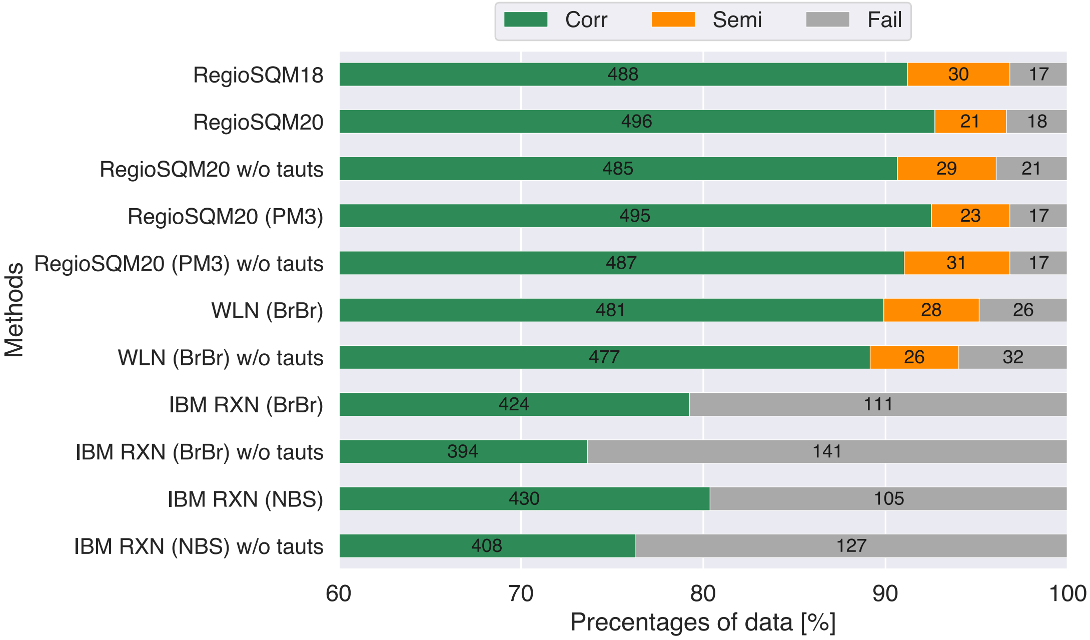


Figure S6: A comparison of different methods used to predict the regioselectivity of the 535 molecules in the data set. "w/o tauts" indicates that the tautomer enumerator is not applied, hence, the results are solely based on the input SMILES. Therefore, "RegioSQM20 w/o tauts" corresponds to "RegioSQM20*" in the paper.

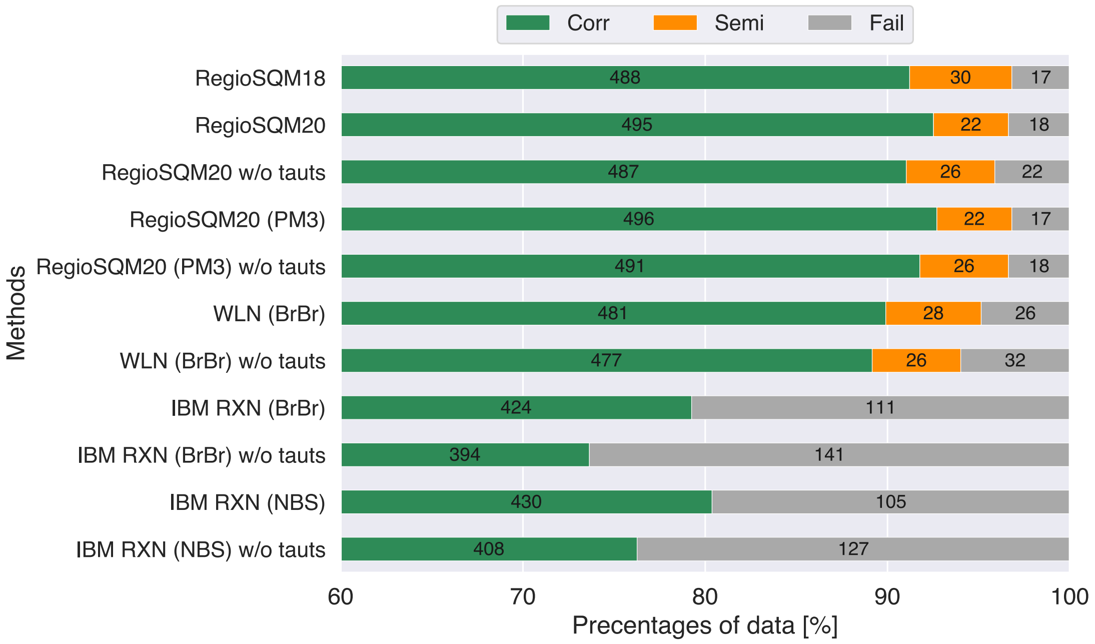


Figure S7: A comparison of different methods without the initial GFNFF-xTB conformer search, which is otherwise used in the four different RegioSQM20 methods.

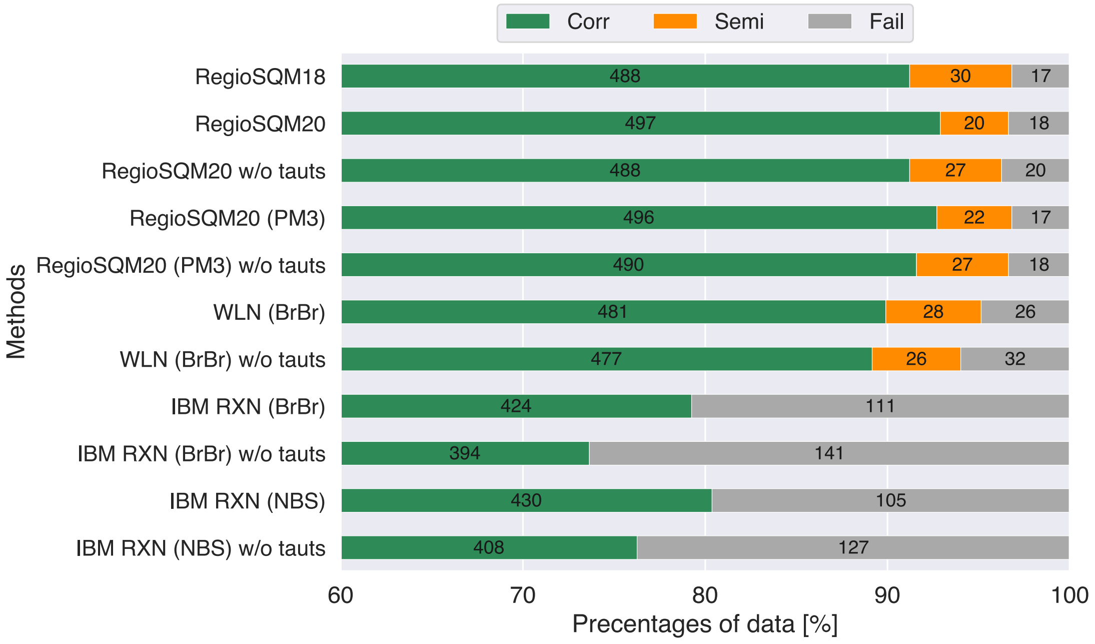


Figure S8: A comparison of different methods with a conformer cutoff of 1000 kcal/mol instead of the default 3 kcal/mol, which is otherwise used in the four different RegioSQM20 methods.

Table S1: The top-1 accuracy of different methods for compounds with one unique black dot[a] and one unique green dot w.r.t. RegioSQM20 w/o tauts (426 out of the 535 compounds in the data set). The results include only the lowest energy tautomer or the tautomer with the overall best atom/confident score.

| Name | 1 site (%) |
|---|---|
| RegioSQM20 | 91.5 |
| RegioSQM20 w/o tauts | 92.0 |
| WLN (BrBr) | 93.2 |
| WLN (BrBr) w/o tauts | 96.0 |
| IBM RXN (BrBr) | 81.7 |
| IBM RXN (BrBr) w/o tauts | 81.9 |
| IBM RXN (NBS) | 84.7 |
| IBM RXN (NBS) w/o tauts | 85.0 |

[a]Out of 535 compounds in the data set, 497 compounds have one unique black dot (experimentally observed reaction site), 37 compounds have two unique black dots, and one compound has three unique black dots.

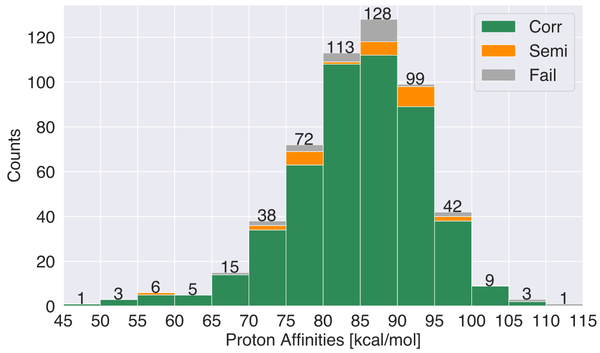

(a) RegioSQM20
Corr: 483, Semi: 27, Fail 25

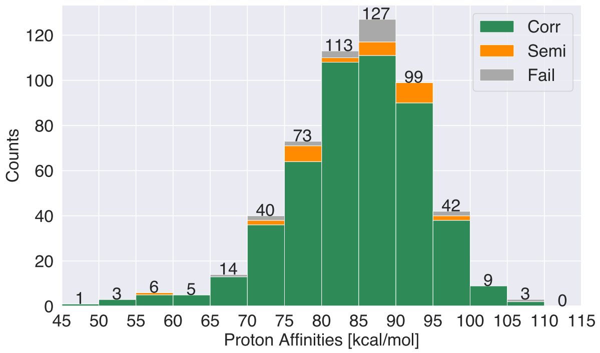

(b) RegioSQM20 w/o tauts
Corr: 485, Semi: 29, Fail 21

Figure S9: Histograms of the highest proton affinities for the 535 molecules in the data set using (a) RegioSQM20 and (b) RegioSQM20 w/o tauts. The values are given in units of kcal/mol. For the RegioSQM20 results, only the lowest energy tautomer of each molecule is considered.

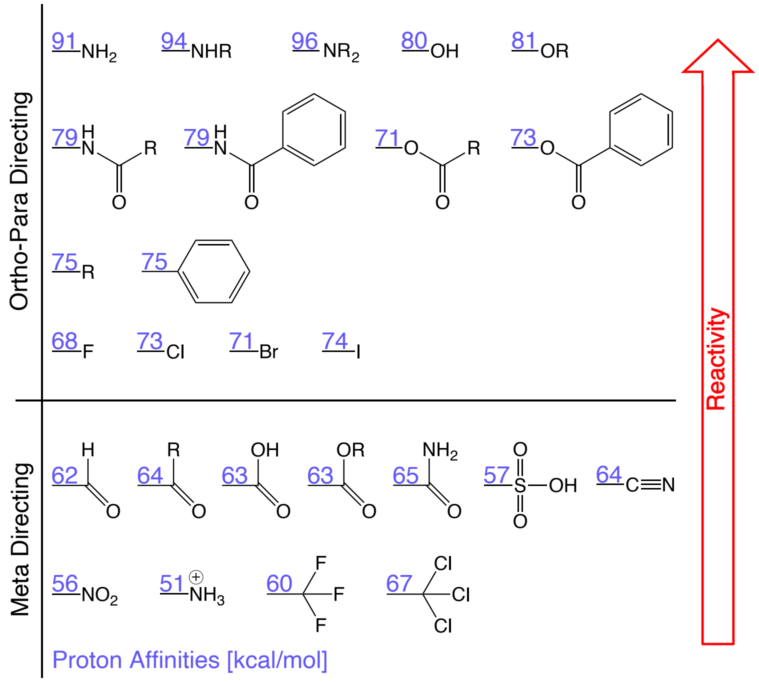

Figure S10: The highest proton affinities computed for a series of substituted benzene analogs using RegioSQM20. The values are given in units of kcal/mol and only the lowest energy tautomer of each molecule is considered.

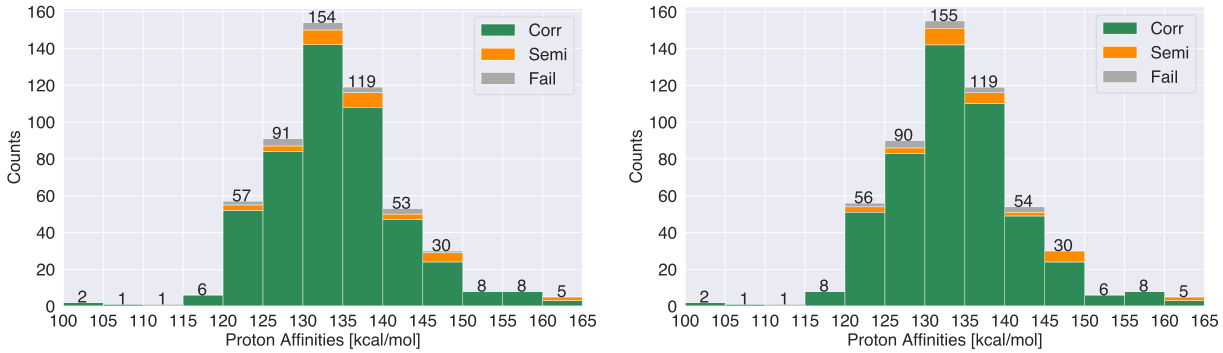


(a) RegioSQM20 (PM3)
Corr: 485, Semi: 32, Fail 18

(b) RegioSQM20 (PM3) w/o tauts
Corr: 487, Semi: 31, Fail 17

Figure S11: Histograms showing the difference in heat of formation between the lowest energy protonated form and the unprotonated form of each molecule in the data set using (a) RegioSQM20 (PM3) and (b) RegioSQM20 (PM3) w/o tauts. The energies are given in units of kcal/mol. For the RegioSQM20 (PM3) results, only the lowest energy tautomer of each molecule is considered.

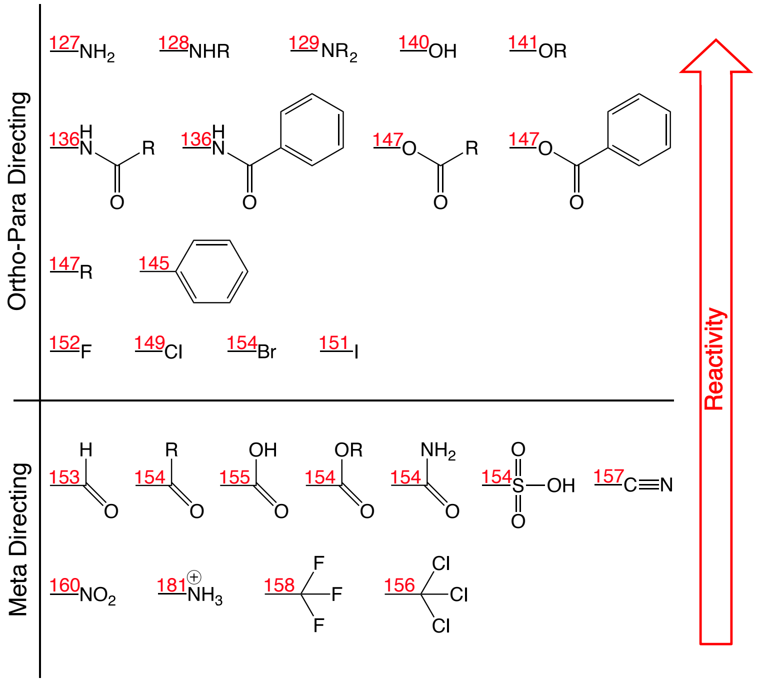


Figure S12: The difference in heat of formation between the lowest energy protonated form and the unprotonated form of each molecule in a series of substituted benzene analogs using RegioSQM20 (PM3). The energies are given in units of kcal/mol and only the lowest energy tautomer of each molecule is considered. Note, the reactivity increases with lower values which is the opposite of Fig. S10, since the heat of formation of the proton is neglected in the values presented here.

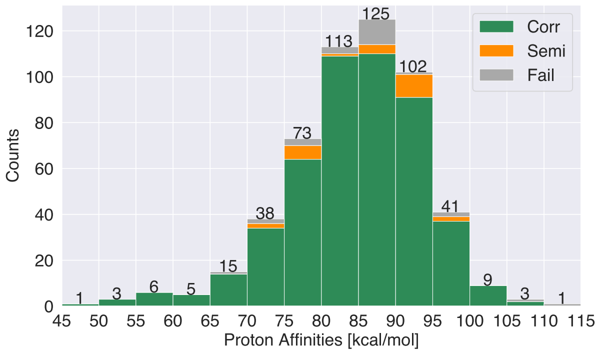


(a) RegioSQM20
Corr: 485, Semi: 25, Fail 25

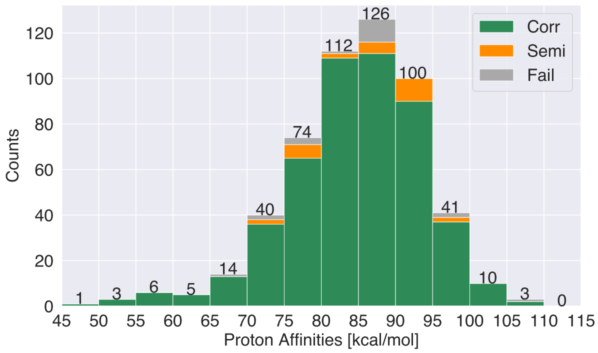


(b) RegioSQM20 w/o tauts
Corr: 488, Semi: 27, Fail 20

Figure S13: Histograms of the highest proton affinities using (a) RegioSQM20 and (b) RegioSQM20 w/o tauts with a conformer cutoff of 1000 kcal/mol instead of the default 3 kcal/mol. For the RegioSQM20 results, only the lowest energy tautomer of each molecule is considered.

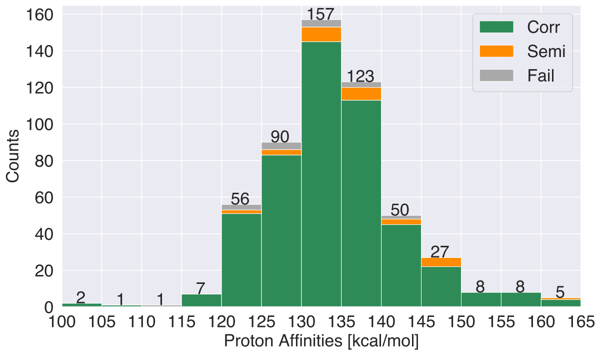


(a) RegioSQM20 (PM3)
Corr: 489, Semi: 29, Fail 17

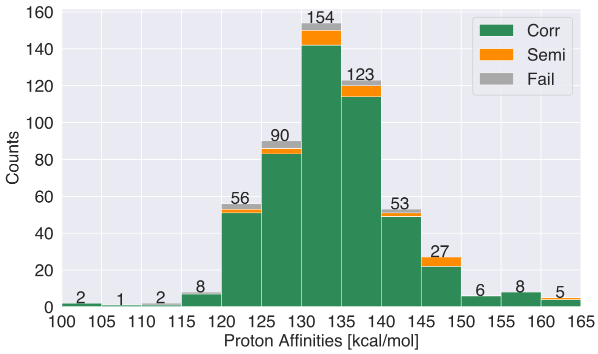


(b) RegioSQM20 (PM3) w/o tauts
Corr: 490, Semi: 27, Fail 18

Figure S14: Histograms showing the difference in heat of formation between the lowest energy protonated form and the unprotonated form of each molecule in the data set using (a) RegioSQM20 (PM3) and (b) RegioSQM20 (PM3) w/o tauts with a conformer cutoff of 1000 kcal/mol instead of the default 3 kcal/mol. The energies are given in units of kcal/mol. For the RegioSQM20 (PM3) results, only the lowest energy tautomer of each molecule is considered.

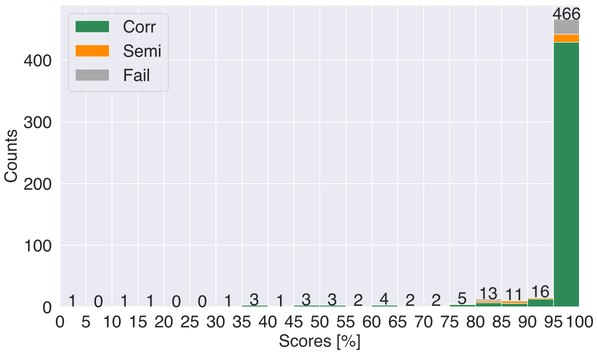


(a) WLN (BrBr) w/o tauts
Corr: 477, Semi: 26, Fail 32

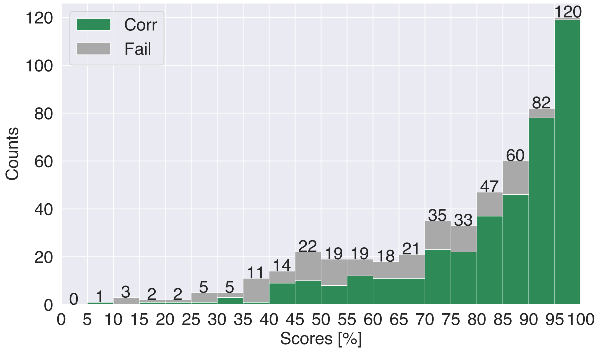


(b) IBM RXN (BrBr) w/o tauts
Corr: 394, Fail 125 (not completed: 16)

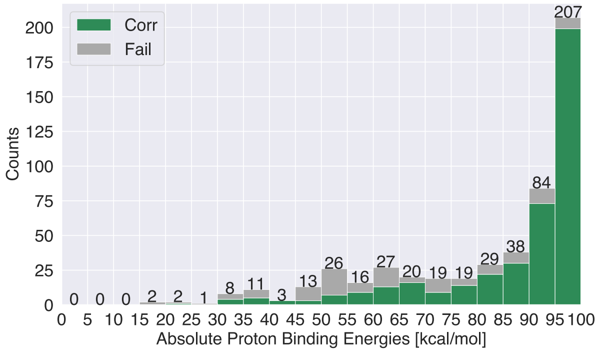


(c) IBM RXN (NBS) w/o tauts
Corr: 408, Fail 117 (not completed: 10)

Figure S15: Histograms showing (a) the best atom score using WLN (BrBr), (b) the confident score using IBM RXN (BrBr), and (c) the confident score using IBM RXN (NBS). All results are solely based on the input SMILES and do not include different tautomers.

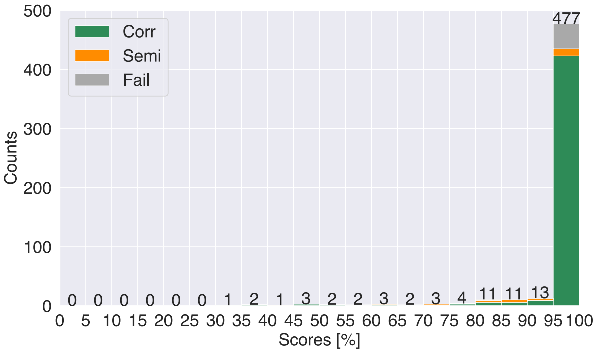


(a) WLN (BrBr).
Corr: 460, Semi: 27, Fail 48

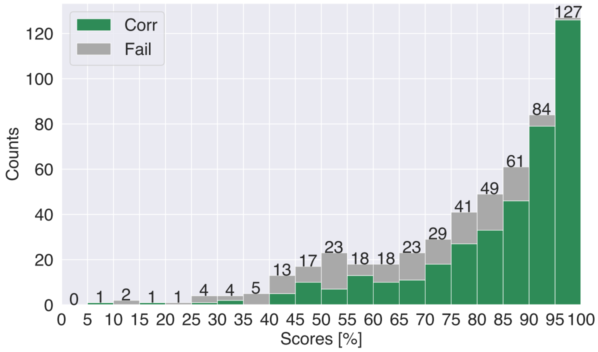


(b) IBM RXN (BrBr).
Corr: 390, Fail 131 (not completed: 14)

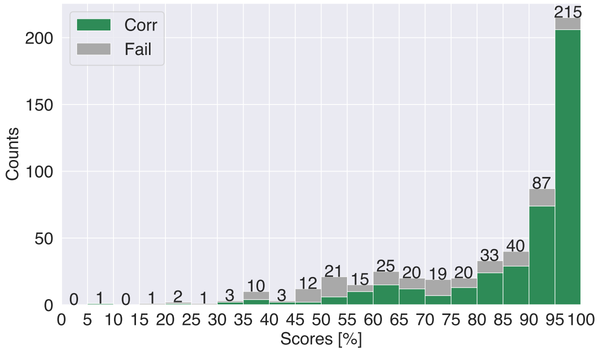


(c) IBM RXN (NBS).
Corr: 408, Fail 120 (not completed: 7)

Figure S16: Histograms showing (a) the best atom score among the tautomers using WLN (BrBr), (b) the best confident score among the tautomers using IBM RXN (BrBr), and (c) the best confident score among the tautomers using IBM RXN (NBS).

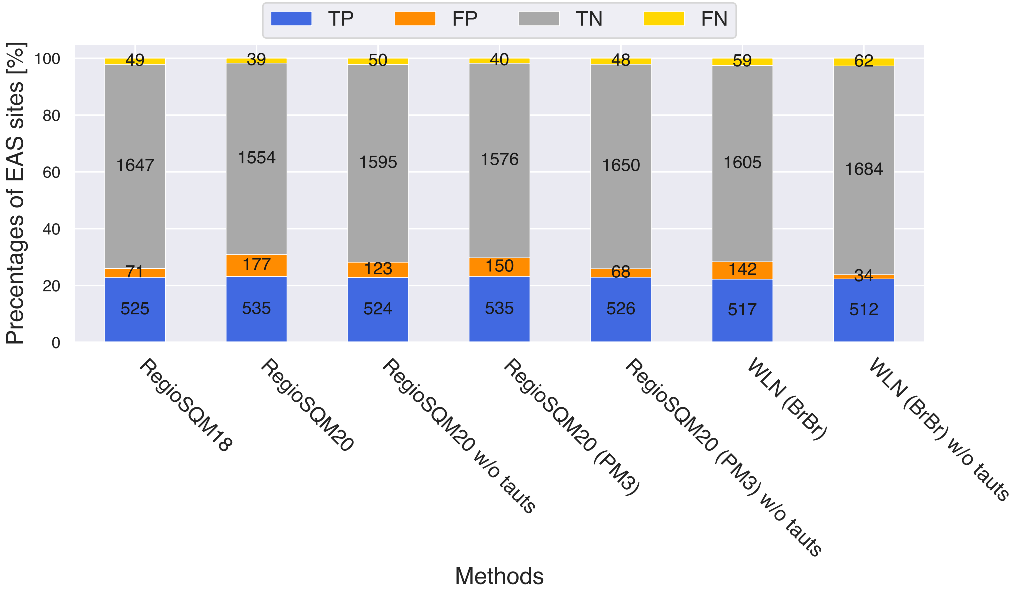


Figure S17: Confusion matrix of all possible reaction sites in the data set. A positive result corresponds to a reaction site being classified as "**Corr**" for any of the tautomers below 15 kcal/mol w.r.t. the RegioSQM methods or all of the generated tautomers w.r.t. the WLN methods. Only unique reaction centers are taken into account.

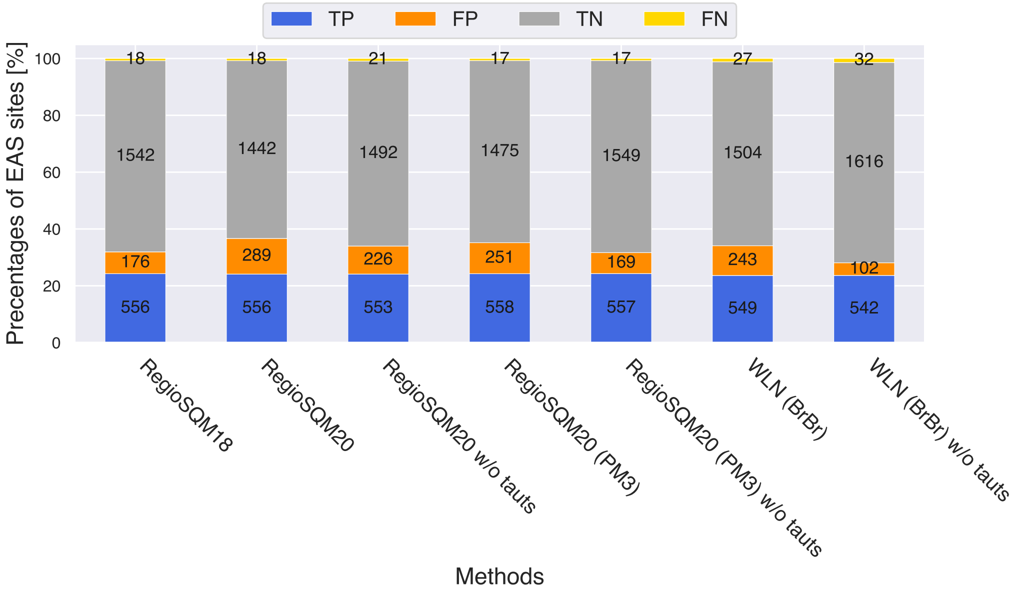


Figure S18: Confusion matrix of all possible reaction sites in the data set. A positive result corresponds to a reaction site being classified as "**Semi**" for any of the tautomers below 15 kcal/mol w.r.t. the RegioSQM methods or all of the generated tautomers w.r.t. the WLN methods. Only unique reaction centers are taken into account.

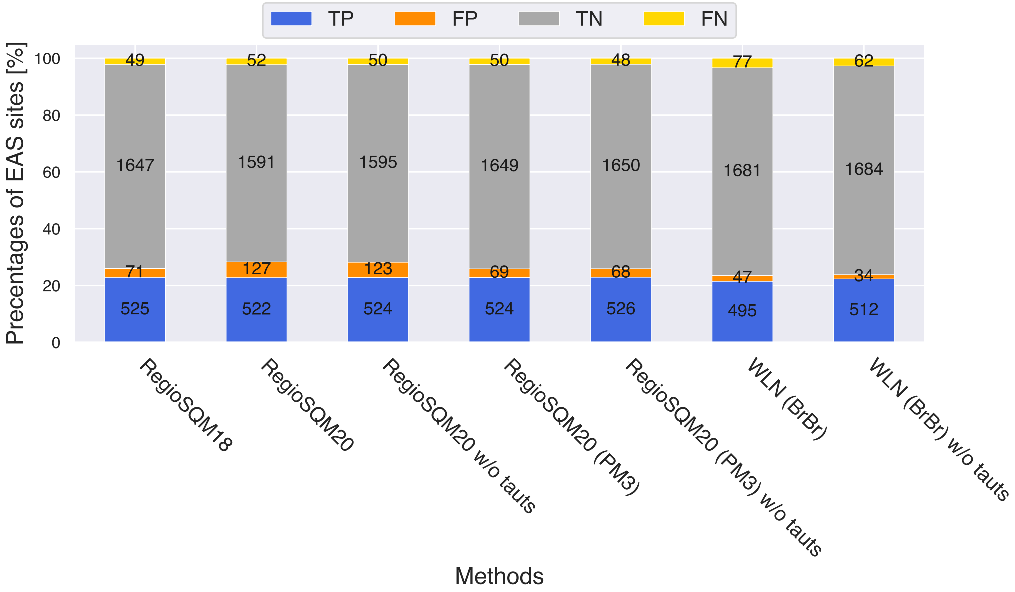


Figure S19: Confusion matrix of all possible reaction sites in the data set. A positive result corresponds to a reaction site being classified as "**Corr**" for only the lowest energy unprotonated tautomer w.r.t. the RegioSQM methods or the tautomer with the overall best atom score w.r.t. the WLN methods. Only unique reaction centers are taken into account.

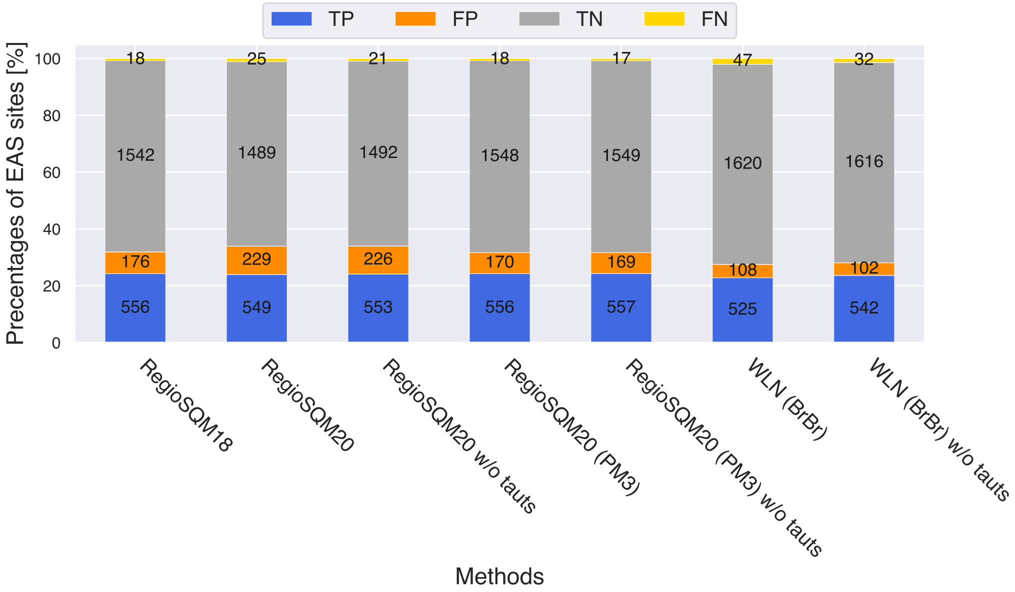


Figure S20: Confusion matrix of all possible reaction sites in the data set. A positive result corresponds to a reaction site being classified as "**Semi**" for only the lowest energy unprotonated tautomer w.r.t. the RegioSQM methods or the tautomer with the overall best atom score w.r.t. the WLN methods. Only unique reaction centers are taken into account.

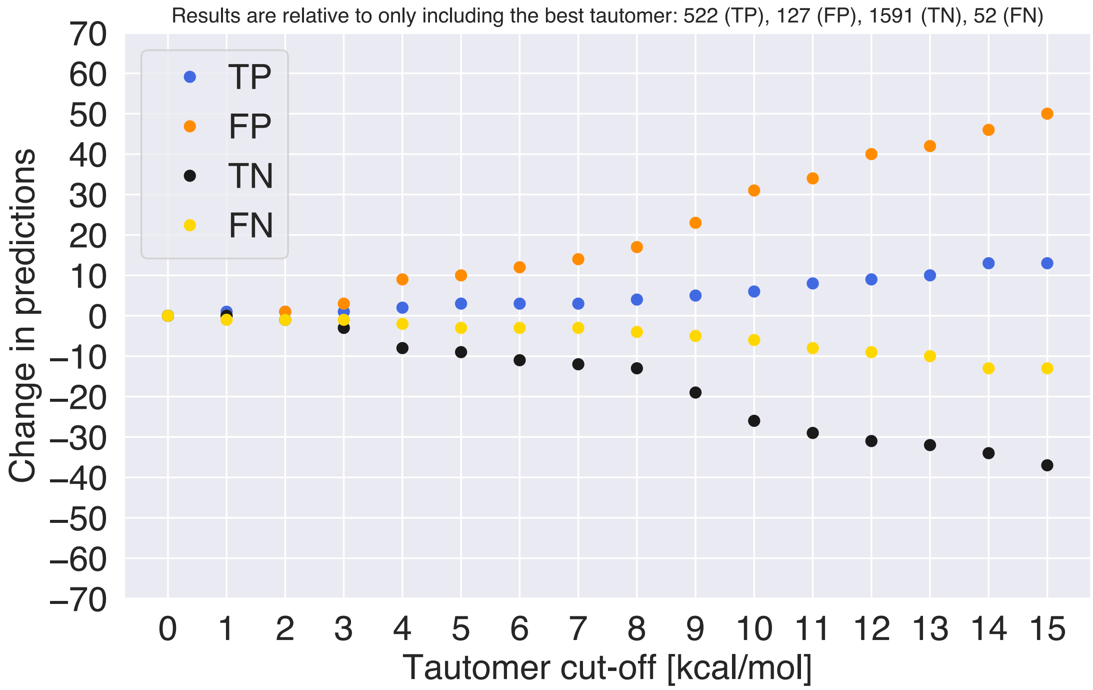


Figure S21: RegioSQM20. Confusion matrix - Corr

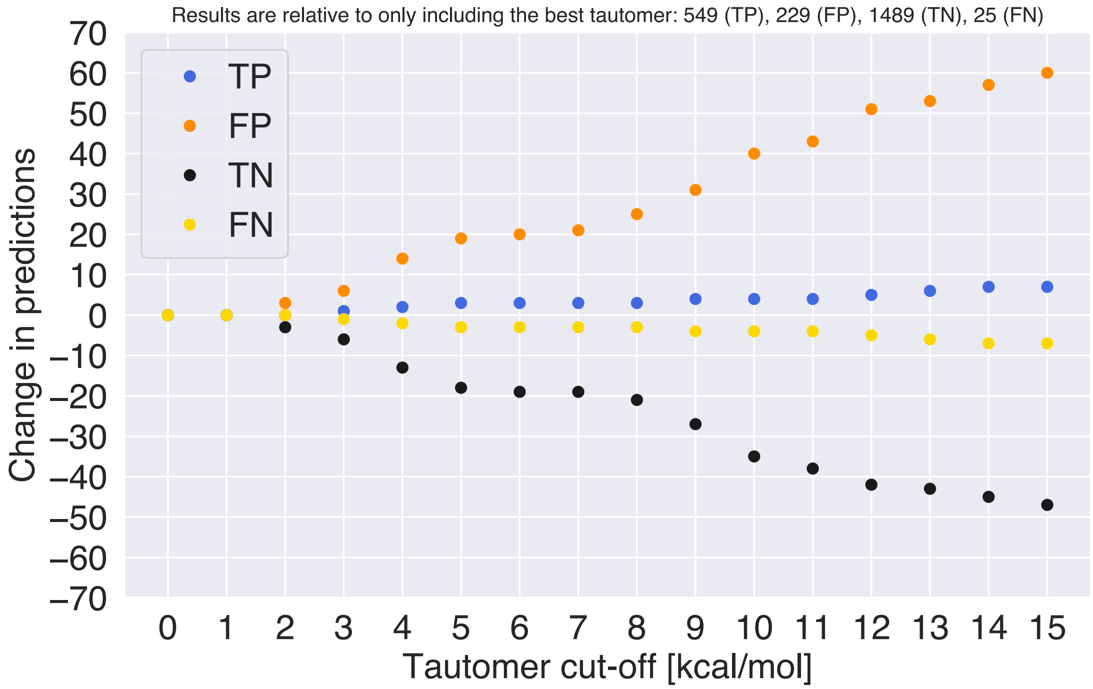


Figure S22: RegioSQM20. Confusion matrix - Semi

# Predictions using RegioSQM20 for the 535 molecules in the data set

# Table of Contents


| | |
|---|---|
| **Pyrroles** | **34** |
| **Furans** | **34** |
| **Thiophenes** | **35** |
| **Pyrazoles** | **36** |
| **Imidazoles** | **37** |
| **Isoxazoles** | **38** |
| **Oxazoles** | **38** |
| **Isothiazoles** | **38** |
| **Thiazoles** | **39** |
| **1,2,4-Triazoles** | **39** |
| **Benzenes** | **40** |
| **Pyridines** | **43** |
| **2-Pyridones** | **46** |
| **Pyridazines** | **47** |
| **Pyrimidines** | **48** |
| **Pyrimidin-2(1H)-ones** | **50** |
| **Pyrimidin-4(3H)-ones** | **51** |
| **Pyrazines** | **52** |
| **Pyrazinones** | **54** |
| **4H-Furo[3,2-b]pyrroles** | **54** |
| **4H-Thieno[3,2-b]pyrroles** | **54** |
| **Imidazo[2,1-b][1,3,4]thiadiazoles** | **55** |
| **Indoles** | **55** |
| **Benzofurans** | **55** |
| **Benzo[b]thiophenes** | **56** |
| **Indazoles** | **57** |
| **1H-Benzo[d]imidazoles** | **58** |

| | |
|---|---|
| **7-Azaindoles** | **59** |
| **6-Azaindoles** | **60** |
| **5-Azaindoles** | **60** |
| **4-Azaindoles** | **61** |
| **Imidazo[1,2-a]pyridines** | **62** |
| **Furo[2,3-b]pyridines** | **62** |
| **Furo[2,3-c]pyridines** | **63** |
| **Furo[3,2-c]pyridines** | **63** |
| **Furo[3,2-b]pyridines** | **63** |
| **[1,2,4]Triazolo[4,3-a]pyridines** | **64** |
| **1H-Pyrazolo[3,4-b]pyridines** | **64** |
| **1H-Imidazo[4,5-c]pyridines** | **64** |
| **Imidazo[1,2-a]pyrazines** | **64** |
| **Imidazo[1,2-c]pyrimidines** | **65** |
| **Imidazo[1,2-b]pyridazines** | **65** |
| **7H-Pyrrolo[2,3-d]pyrimidines** | **65** |
| **Pyrazolo[1,5-c]pyrimidines** | **65** |
| **Imidazo[1,5-a]pyrazines** | **66** |
| **Pyrrolo[2,1-f][1,2,4]triazines** | **66** |
| **Oxazolo[4,5-b]pyridines** | **66** |
| **Furo[2,3-d]pyrimidines** | **66** |
| **Furo[2,3-b]pyrazines** | **67** |
| **[1,2,4]Triazolo[4,3-b]pyridazines** | **67** |
| **Pyrazolo[1,5-a][1,3,5]triazines** | **67** |
| **Naphthalenes** | **68** |
| **Quinolines** | **68** |
| **Isoquinolines** | **68** |

Quinoxalines 68

1,5-Naphthyridines 69

Pyrido[4,3-d]pyrimidines 69

Pyrido[3,4-b]pyrazines 69

Furo[3,2-c]pyridin-4(5H)-ones 69

Furo[2,3-d]pyrimidin-4(3H)-ones 70

Imidazo[1,2-a]pyrazin-8(7H)-ones 70

Thiazolo[5,4-b]pyridin-5(4H)-ones 70

3,4-Dihydro-5H-[1,2,3]triazolo[4,5-b]pyridin-5-ones 70

1,3-Dihydro-2H-benzo[d]imidazol-2-ones 71

Oxazolo[4,5-b]pyridin-2(3H)-ones 71

Quinazolin-4(3H)-ones 71

1,7-Naphthyridin-8(7H)-ones 71

Pyrido[2,3-d]pyrimidin-7(8H)-ones 72

2H-Chromen-2-ones 72

# Pyrroles

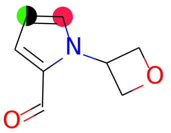

Medium | 0.00

(S23.1) **402**

# Furans

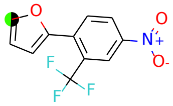

Medium | 0.00

(S24.1) **434**

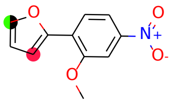

Medium | 0.00

(S24.2) **435**

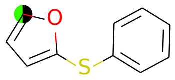

Medium | 0.00

(S24.3) **437**

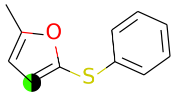

Medium | 0.00

(S24.4) **438**

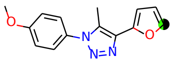

Medium | 0.00

(S24.5) **441**

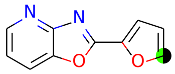

Medium | 0.00

(S24.6) **442**

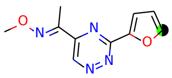

Medium | 0.00

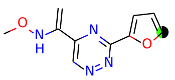

Medium | 5.68

(S24.7) **444**

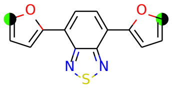

Medium | 0.00

(S24.8) **446**

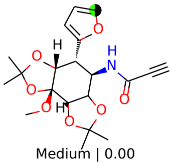

Medium | 0.00

(S24.9) **449**

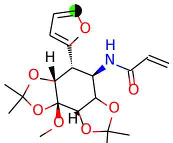

Medium | 0.00

(S24.10) **450**

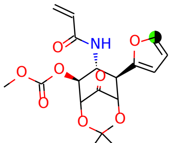

Medium | 0.00

(S24.11) **451**

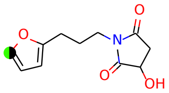

Medium | 0.00

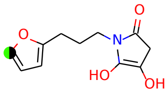

Medium | 11.85

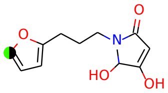

Medium | 12.47

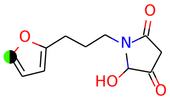

Medium | 13.26

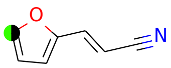

Medium | 0.00

(S24.12) **452**

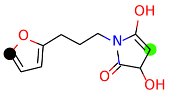

High | 14.22

(S24.13) **453**

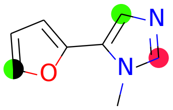

Medium | 0.00

(S24.14) **454**

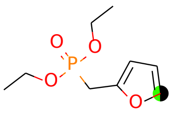

Medium | 0.00

(S24.15) **465**

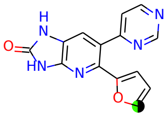

Medium | 0.00

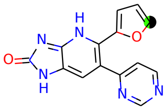

Medium | 8.40

(S24.16) **466**

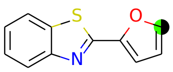

Medium | 0.00

(S24.17) **469**

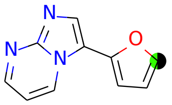

Medium | 0.00

(S24.18) **474**

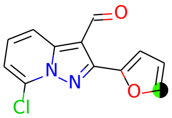

Medium | 0.00

(S24.19) **476**

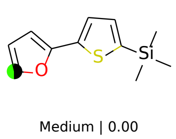


(S24.20) **477**

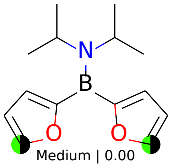


(S24.21) **478**

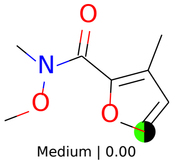


(S24.22) **479**

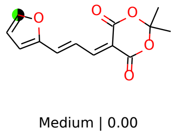


(S24.23) **483**

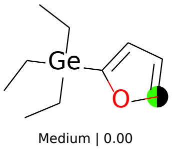


(S24.24) **484**

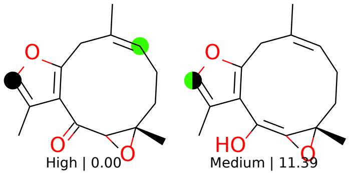


(S24.25) **485**

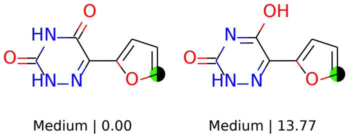


(S24.26) **486**

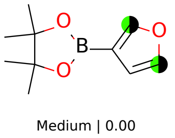


(S24.27) **443**

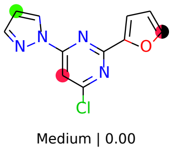


(S24.28) **475**

# Thiophenes

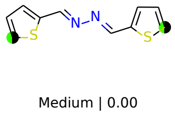


(S25.1) **413**

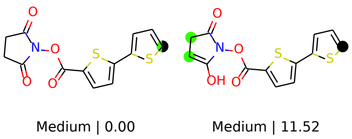


(S25.2) **586**

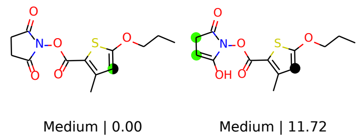


(S25.3) **588**

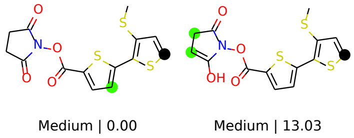


(S25.4) **587**

# Pyrazoles

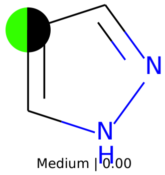


(S26.1) **1**

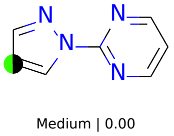


(S26.2) **2**

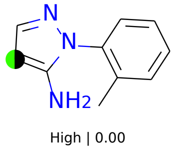


(S26.3) **3**

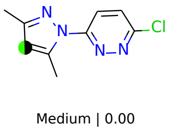


(S26.4) **4**

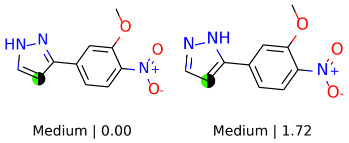


(S26.5) **5**

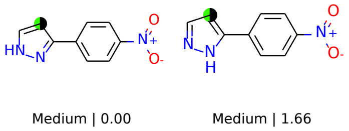


(S26.6) **50**

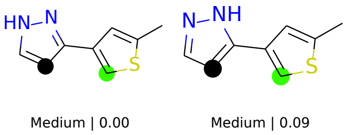


(S26.7) **51**

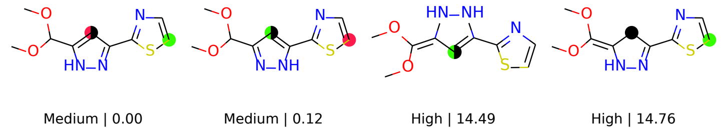


(S26.8) **52**

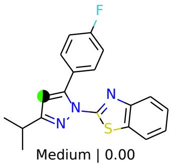


(S26.9) **53**

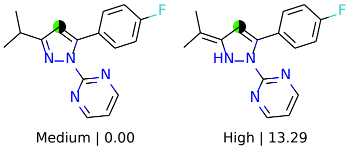


(S26.10) **54**

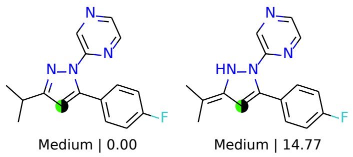


(S26.11) **55**

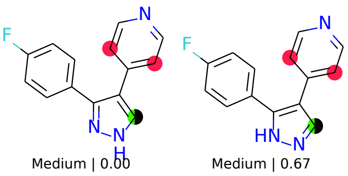


(S26.12) **56**

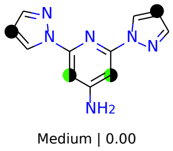


(S26.13) **216**

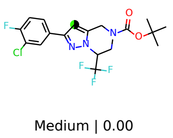


(S26.14) **278**

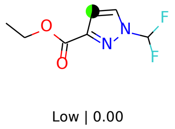


(S26.15) **392**

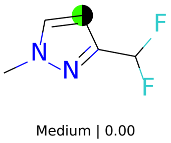


(S26.16) **397**

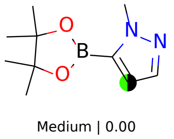


(S26.17) **421**

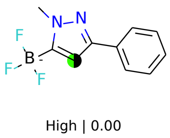


(S26.18) **599**

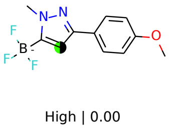


(S26.19) **600**

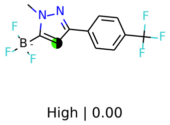


(S26.20) **601**

# Imidazoles

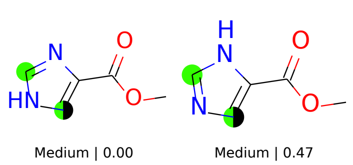


(S27.1) **14**

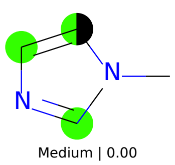


(S27.2) **15**

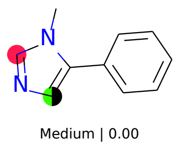


(S27.3) **16**

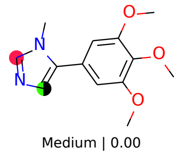


(S27.4) **17**

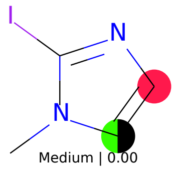


(S27.5) **58**

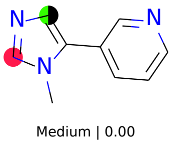


(S27.6) **125**

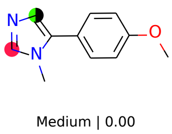


(S27.7) **126**

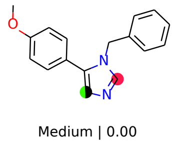


(S27.8) **127**

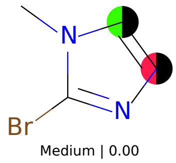


(S27.9) **155**

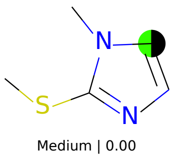


(S27.10) **156**

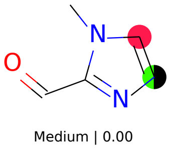


(S27.11) **157**

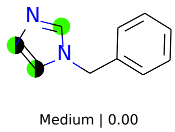


(S27.12) **158**

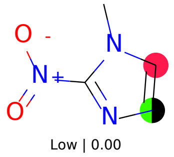


(S27.13) **160**

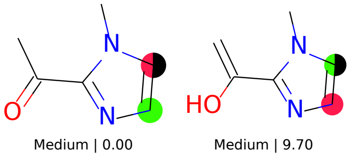


(S27.14) **161**

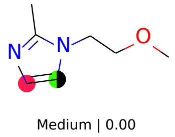


(S27.15) **162**

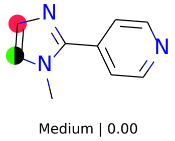


(S27.16) **165**

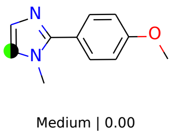


(S27.17) **166**

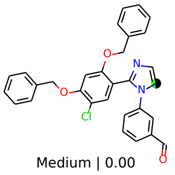


(S27.18) **171**

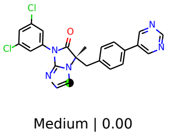


(S27.19) **173**

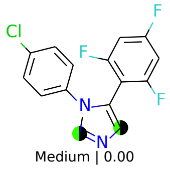


(S27.20) **137**

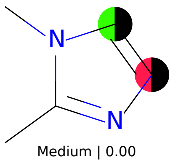


(S27.21) **154**

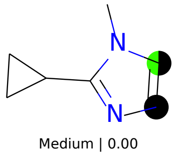


(S27.22) **159**

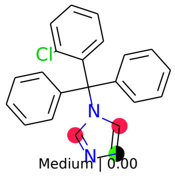


(S27.23) **163**

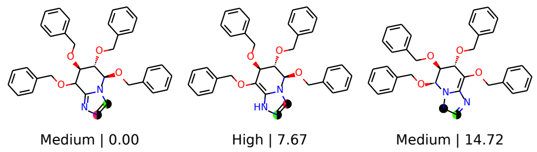


(S27.24) **164**

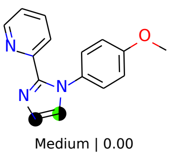


(S27.25) **168**

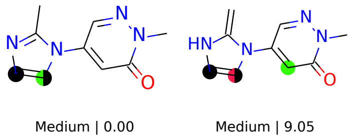


(S27.26) **169**

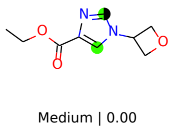


(S27.27) **401**

# Isoxazoles

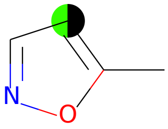
Medium | 0.00
(S28.1) **6**

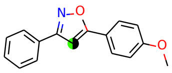
Medium | 0.00
(S28.2) **7**

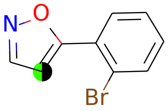
Medium | 0.00
(S28.3) **8**

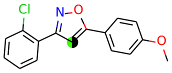
Medium | 0.00
(S28.4) **9**

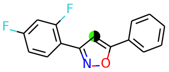
Medium | 0.00
(S28.5) **141**

# Oxazoles

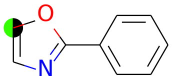
Medium | 0.00
(S29.1) **43**

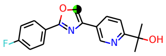
Medium | 0.00
(S29.2) **119**

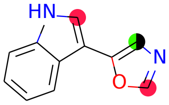
Medium | 0.00
(S29.3) **122**

# Isothiazoles

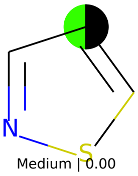
Medium | 0.00
(S30.1) **10**

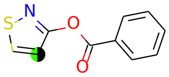
Medium | 0.00
(S30.2) **11**

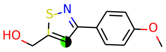
Medium | 0.00
(S30.3) **13**

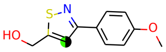
Medium | 0.00
(S30.4) **598**

# Thiazoles

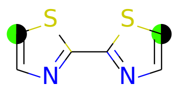

Medium | 0.00

(S31.1) **18**

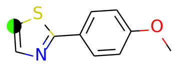

Medium | 0.00

(S31.2) **19**

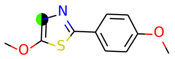

Medium | 0.00

(S31.3) **20**

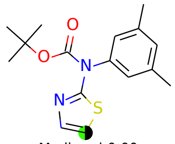

Medium | 0.00

(S31.4) **59**

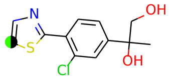

Medium | 0.00

(S31.5) **60**

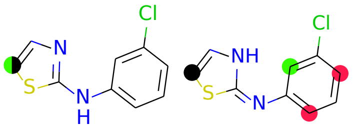

Medium | 0.00 Medium | 4.70

(S31.6) **61**

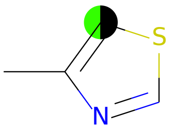

Medium | 0.00

(S31.7) **62**

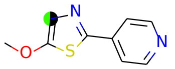

Medium | 0.00

(S31.8) **64**

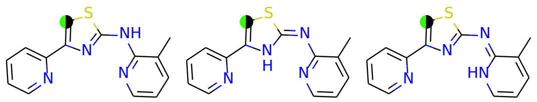

Medium | 0.00 Medium | 1.78 High | 6.53

(S31.9) **66**

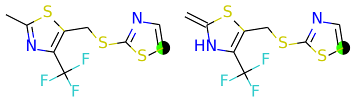

Medium | 0.00 Medium | 11.64

(S31.10) **67**

# 1,2,4-Triazoles

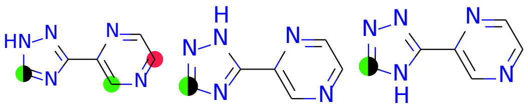

Low | 0.00 Low | 0.92 Medium | 1.52

(S32.1) **147**

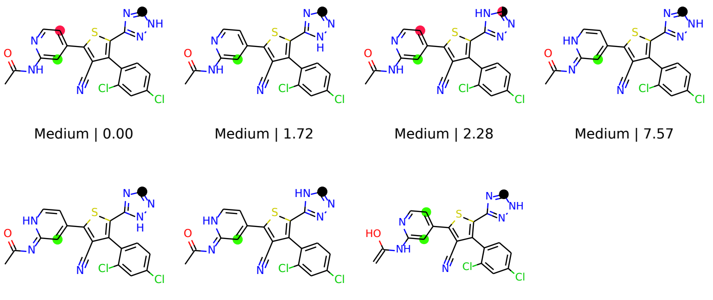

Medium | 0.00 Medium | 1.72 Medium | 2.28 Medium | 7.57

Medium | 9.00 Medium | 9.32 Medium | 14.48

(S32.2) **149**

# Benzenes

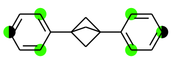

Medium | 0.00

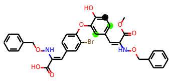

Medium | 0.00

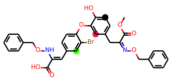

Medium | 3.91

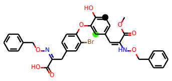

Medium | 4.24

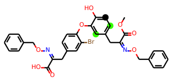

Medium | 11.76

(S33.1) **274** (S33.2) **276**

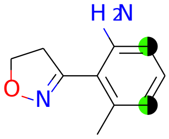

Medium | 0.00

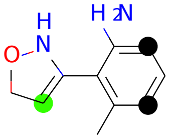

High | 6.54

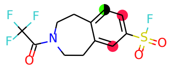

Low | 0.00

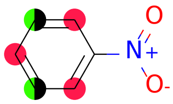

Low | 0.00

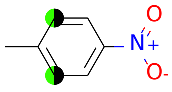

Low | 0.00

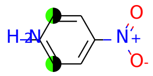

Medium | 0.00

(S33.3) **277** (S33.4) **428** (S33.5) **488** (S33.6) **489** (S33.7) **490**

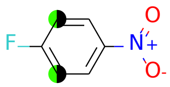

Low | 0.00

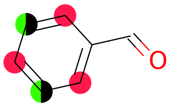

Low | 0.00

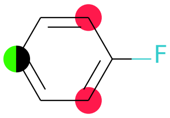

Low | 0.00

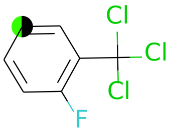

Low | 0.00

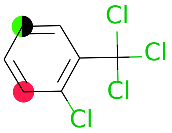

Low | 0.00

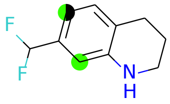

Medium | 0.00

(S33.8) **493** (S33.9) **494** (S33.10) **495** (S33.11) **496** (S33.12) **497** (S33.13) **498**

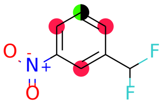

Low | 0.00

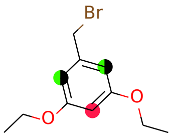

Medium | 0.00

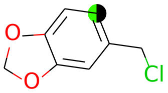

Medium | 0.00

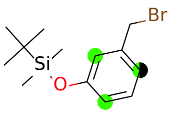

Medium | 0.00

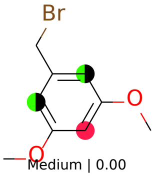

Medium | 0.00

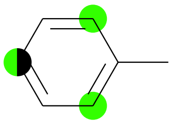

Medium | 0.00

(S33.14) **499** (S33.15) **500** (S33.16) **501** (S33.17) **502** (S33.18) **503** (S33.19) **504**

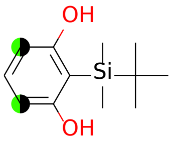

Medium | 0.00

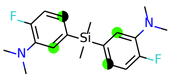

Medium | 0.00

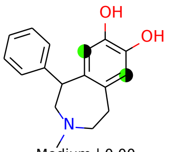

Medium | 0.00

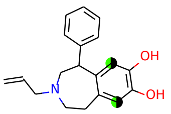

Medium | 0.00

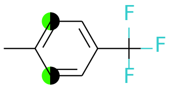

Low | 0.00

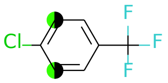

Low | 0.00

(S33.20) **509** (S33.21) **511** (S33.22) **514** (S33.23) **515** (S33.24) **516** (S33.25) **517**

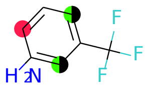

Medium | 0.00

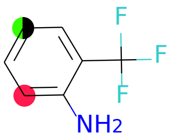

Medium | 0.00

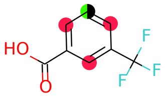

Low | 0.00

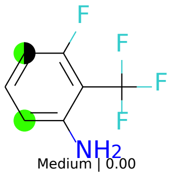

Medium | 0.00

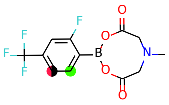

Low | 0.00

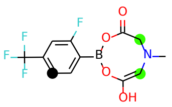

Medium | 7.67

(S33.26) **518** (S33.27) **519** (S33.28) **520** (S33.29) **521** (S33.30) **522**

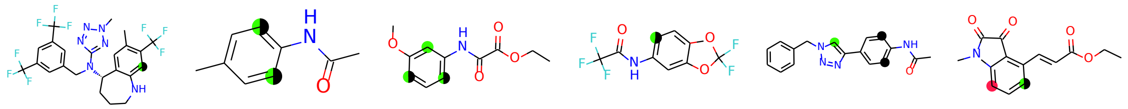


(S33.31) **523** (S33.32) **526** (S33.33) **529** (S33.34) **530** (S33.35) **531** (S33.36) **532**

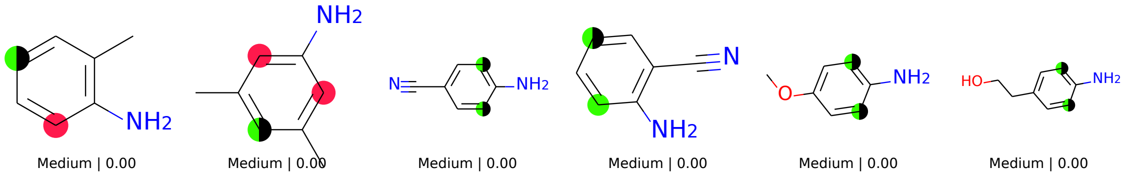


(S33.37) **534** (S33.38) **535** (S33.39) **536** (S33.40) **537** (S33.41) **539** (S33.42) **540**

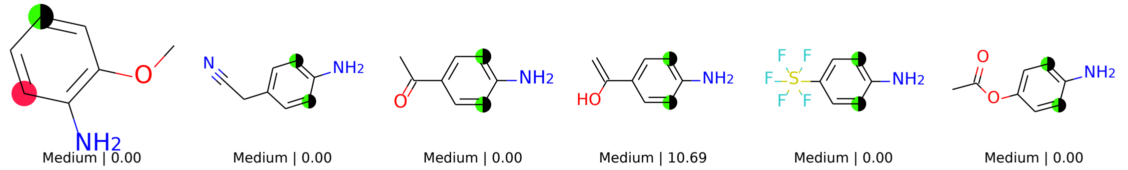


(S33.43) **541** (S33.44) **542** (S33.45) **543** (S33.46) **546** (S33.47) **549**

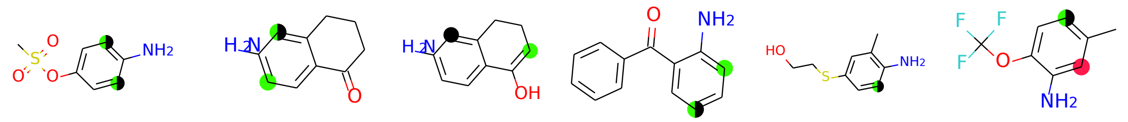


(S33.48) **550** (S33.49) **551** (S33.50) **552** (S33.51) **553** (S33.52) **554**

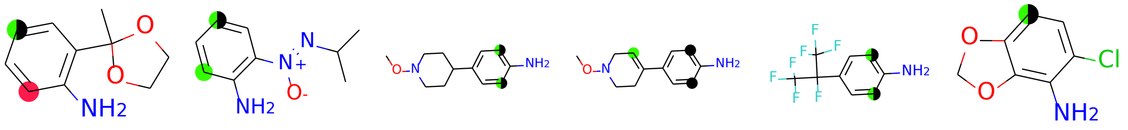


(S33.53) **555** (S33.54) **556** (S33.55) **558** (S33.56) **559** (S33.57) **560** (S33.58) **562**

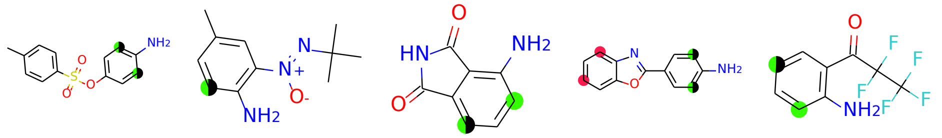


(S33.59) **563** (S33.60) **564** (S33.61) **566** (S33.62) **567** (S33.63) **568**

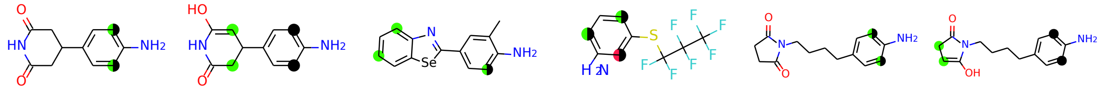


(S33.64) **569** (S33.65) **570** (S33.66) **571** (S33.67) **572**

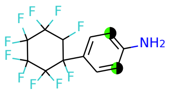

Medium | 0.00

(S33.68) **573**

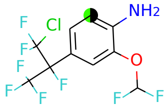

Medium | 0.00

(S33.69) **575**

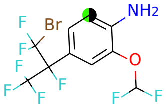

Medium | 0.00

(S33.70) **576**

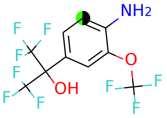

Medium | 0.00

(S33.71) **577**

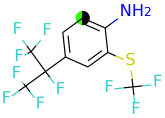

Medium | 0.00

(S33.72) **578**

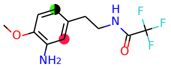

Medium | 0.00

(S33.73) **579**

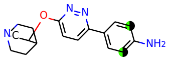

Medium | 0.00

(S33.74) **582**

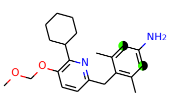

Medium | 0.00

(S33.75) **583**

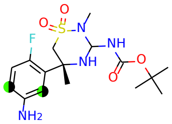

Medium | 0.00

(S33.76) **584**

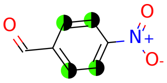

Low | 0.00

(S33.77) **492**

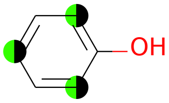

Medium | 0.00

(S33.78) **505**

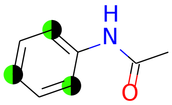

Medium | 0.00

(S33.79) **524**

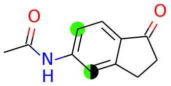

Medium | 0.00

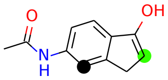

High | 9.19

(S33.80) **528**

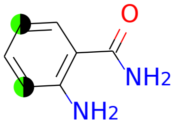

Medium | 0.00

(S33.81) **545**

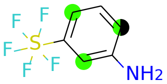

Medium | 0.00

(S33.82) **547**

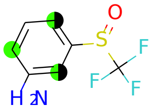

Medium | 0.00

(S33.83) **561**

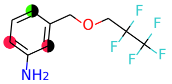

Medium | 0.00

(S33.84) **565**

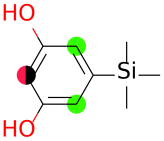

Medium | 0.00

(S33.85) **508**

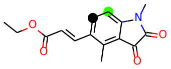

Low | 0.00

(S33.86) **533**

# Pyridines

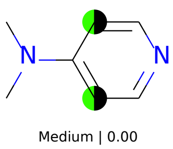


(S34.1) **21**

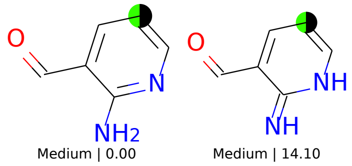


(S34.2) **22**

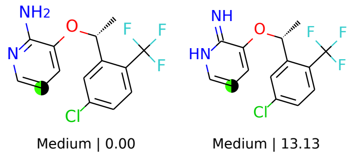


(S34.3) **23**

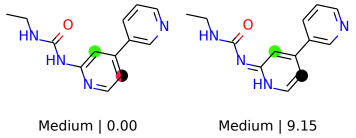


(S34.4) **24**

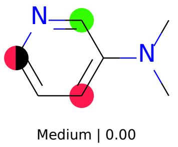


(S34.5) **48**

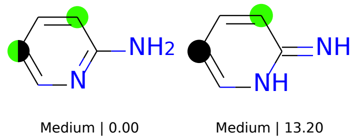


(S34.6) **68**

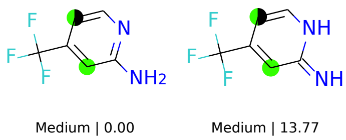


(S34.7) **69**

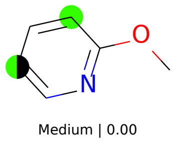


(S34.8) **70**

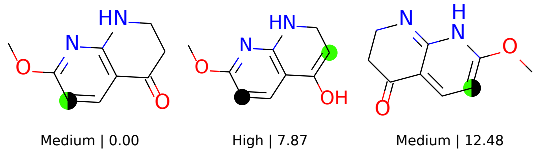


(S34.9) **71**

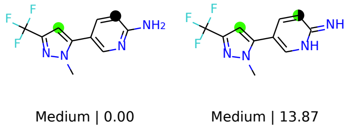


(S34.10) **73**

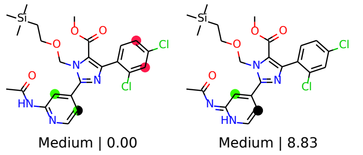


(S34.11) **75**

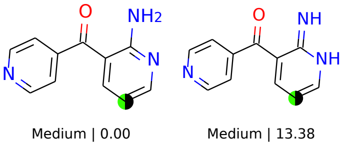


(S34.12) **76**

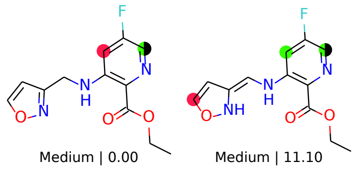


(S34.13) **124**

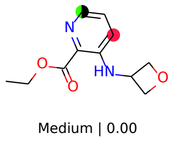


(S34.14) **128**

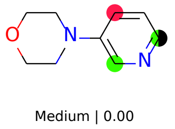


(S34.15) **129**

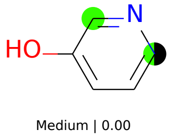


(S34.16) **130**

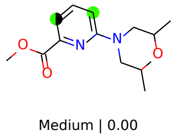


(S34.17) **215**

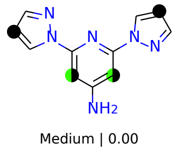


(S34.18) **216**

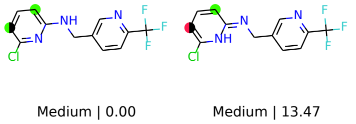


(S34.19) **217**

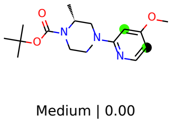


(S34.20) **218**

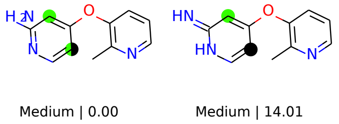


(S34.21) **221**

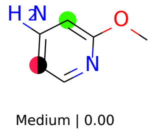


(S34.22) **223**

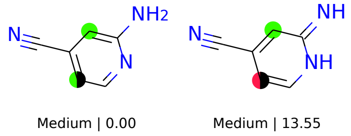


(S34.23) **224**

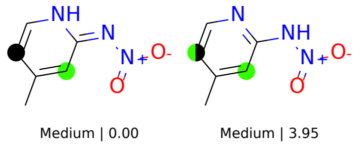


(S34.24) **225**

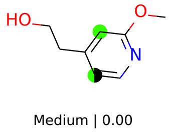


(S34.25) **228**

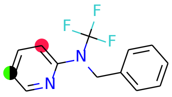

Medium | 0.00

(S34.26) **229**

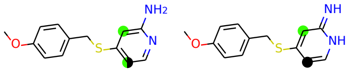

Medium | 0.00 High | 13.34

(S34.27) **234**

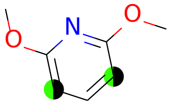

Medium | 0.00

(S34.28) **236**

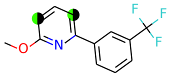

Medium | 0.00

(S34.29) **237**

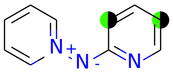

Medium | 0.00

(S34.30) **238**

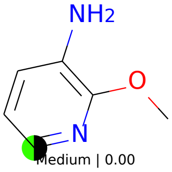

Medium | 0.00

(S34.31) **239**

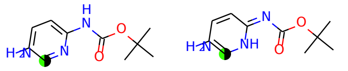

Medium | 0.00 Medium | 7.95

(S34.32) **241**

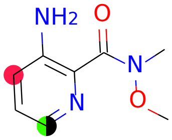

Medium | 0.00

(S34.33) **242**

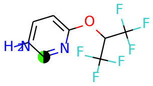

Medium | 0.00

(S34.34) **243**

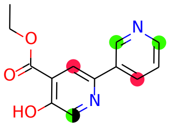

Low | 0.00

(S34.35) **244**

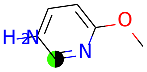

Medium | 0.00

(S34.36) **247**

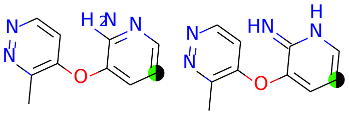

Medium | 0.00 Medium | 10.55

(S34.37) **248**

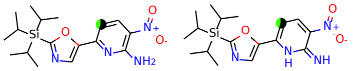

Medium | 0.00 Medium | 14.90

(S34.38) **249**

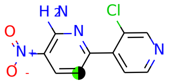

Medium | 0.00

(S34.39) **250**

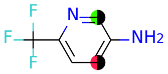

Medium | 0.00

(S34.40) **252**

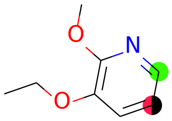

Medium | 0.00

(S34.41) **253**

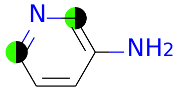

Medium | 0.00

(S34.42) **255**

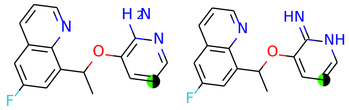

Medium | 0.00 Medium | 13.22

(S34.43) **256**

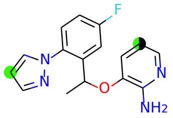

Medium | 0.00

(S34.44) **257**

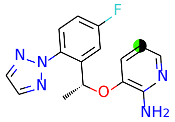

Medium | 0.00

(S34.45) **258**

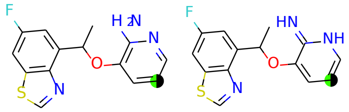

Medium | 0.00 Medium | 13.05

(S34.46) **259**

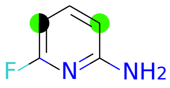

Medium | 0.00

(S34.47) **296**

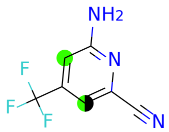

Medium | 0.00

(S34.48) **297**

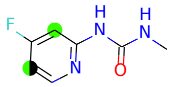

Medium | 0.00 Medium | 7.21

(S34.49) **298**

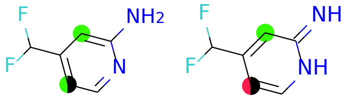

Medium | 0.00 Medium | 13.92

(S34.50) **391**

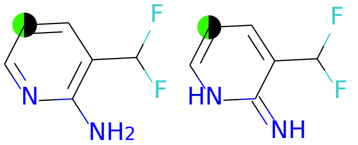

Medium | 0.00 Medium | 12.64

(S34.51) **395**

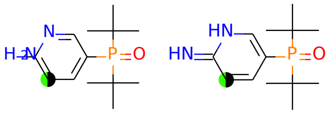

Medium | 0.00 Medium | 13.70

(S34.52) **400**

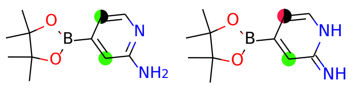

Medium | 0.00 Medium | 12.65

(S34.53) **420**

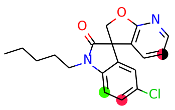

Medium | 0.00

(S34.54) **74**

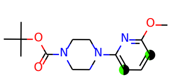

Medium | 0.00

(S34.55) **219**

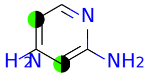

Medium | 0.00

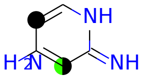

High | 13.37

(S34.56) **220**

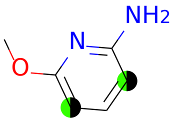

Medium | 0.00

(S34.57) **235**

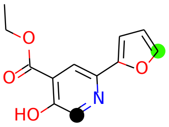

Medium | 0.00

(S34.58) **245**

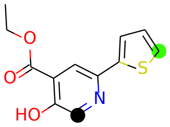

Medium | 0.00

(S34.59) **246**

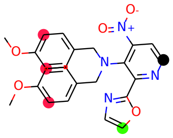

Medium | 0.00

(S34.60) **251**

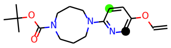

Medium | 0.00

(S34.61) **254**

# 2-Pyridones

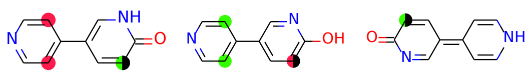

Medium | 0.00 Medium | 8.04 Medium | 13.83

(S35.1) **77**

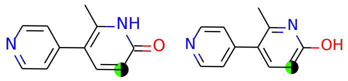

Medium | 0.00 Medium | 8.35

(S35.2) **78**

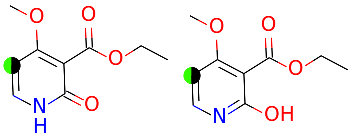

Medium | 0.00 Medium | 6.90

(S35.3) **79**

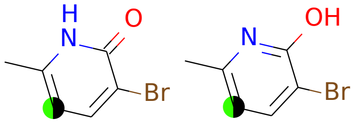

Medium | 0.00 Medium | 8.94

(S35.4) **80**

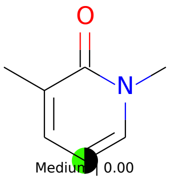

Medium | 0.00

(S35.5) **81**

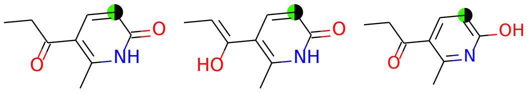

Medium | 0.00 Medium | 8.83 Medium | 8.96

(S35.6) **82**

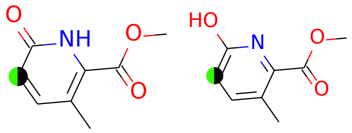

Medium | 0.00 Low | 7.53

(S35.7) **83**

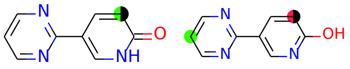

Medium | 0.00 Medium | 8.12

(S35.8) **84**

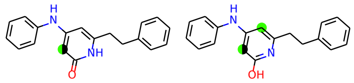

Medium | 0.00 Medium | 9.28

(S35.9) **213**

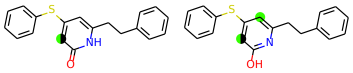

Medium | 0.00 Medium | 9.07

(S35.10) **214**

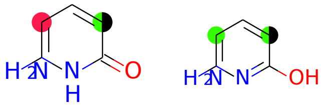

Medium | 0.00 Medium | 8.08

(S35.11) **227**

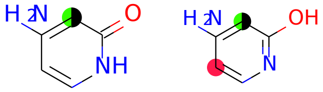

Medium | 0.00 Medium | 9.70

(S35.12) **230**

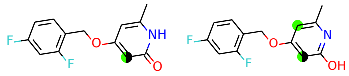

Medium | 0.00 Medium | 10.49

(S35.13) **233**

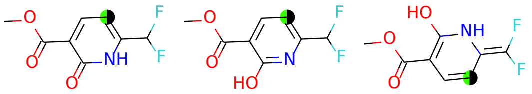

Low | 0.00 Low | 3.56 Medium | 10.39

(S35.14) **398**

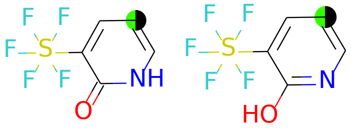

Low | 0.00 Low | 6.29

(S35.15) **427**

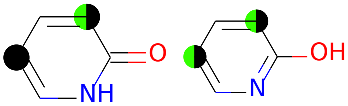

Medium | 0.00 Medium | 8.52

(S35.16) **226**

# Pyridazines

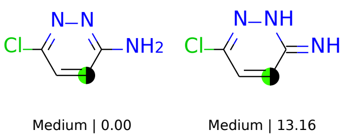


(S36.1) **32**

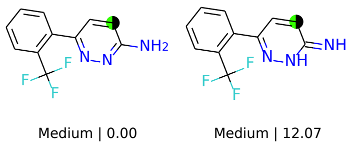


(S36.2) **33**

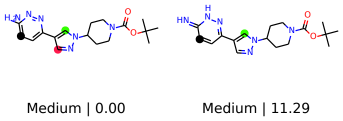


(S36.3) **34**

# Pyrimidines

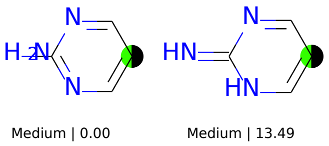


(S37.1) **25**

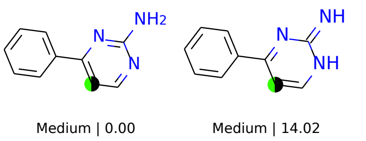


(S37.2) **26**

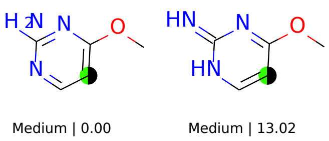


(S37.3) **27**

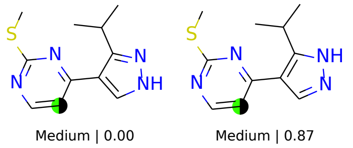


(S37.4) **28**

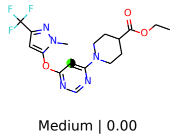


(S37.5) **29**

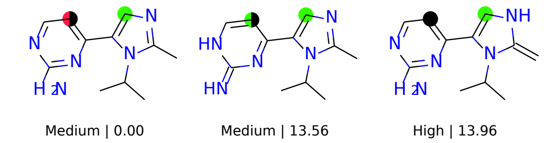


(S37.6) **85**

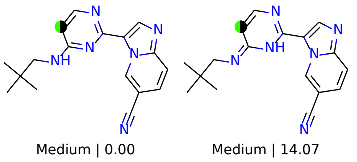


(S37.7) **86**

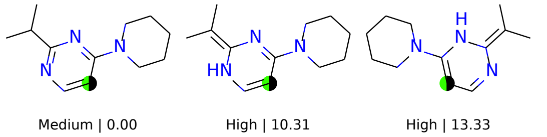


(S37.8) **87**

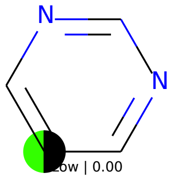


(S37.9) **88**

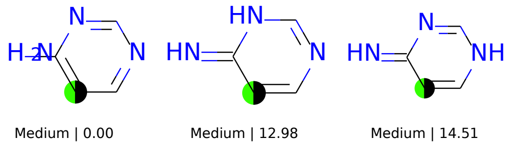


(S37.10) **89**

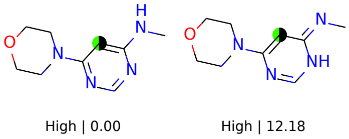


(S37.11) **90**

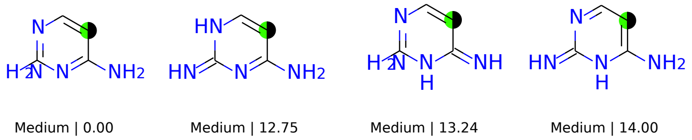


(S37.12) **91**

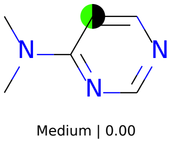


(S37.13) **92**

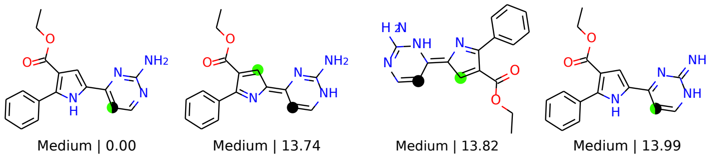


(S37.14) **93**

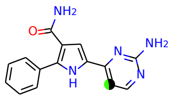
Medium | 0.00

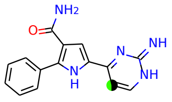
Medium | 13.82

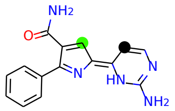
High | 14.23

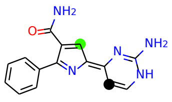
High | 14.74

(S37.15) **94**

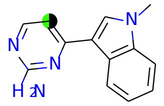
Medium | 0.00

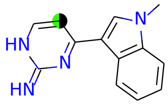
Medium | 13.18

(S37.16) **96**

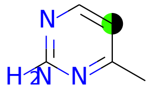
Medium | 0.00

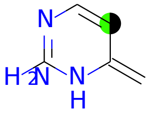
High | 10.17

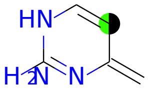
Medium | 12.60

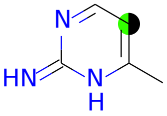
Medium | 13.17

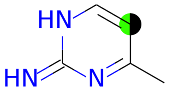
Medium | 13.38

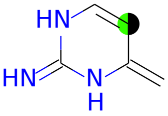
Medium | 14.82

(S37.17) **97**

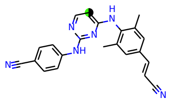
Medium | 0.00

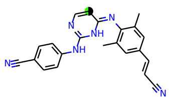
Medium | 9.42

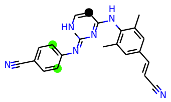
Medium | 10.77

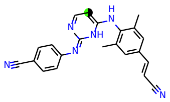
Medium | 12.74

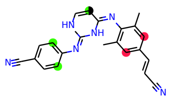
Medium | 13.48

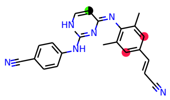
Medium | 13.62

(S37.18) **98**

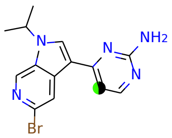
Medium | 0.00

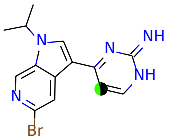
Medium | 13.35

(S37.19) **324**

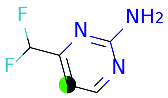
Medium | 0.00

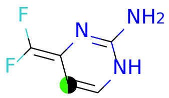
Medium | 8.55

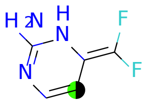
Medium | 10.43

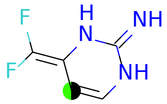
Medium | 14.40

(S37.20) **390**

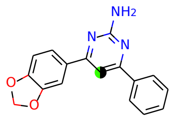
Medium | 0.00

(S37.21) **593**

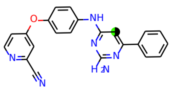
Medium | 0.00

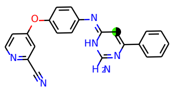
Medium | 10.70

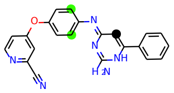
Medium | 12.77

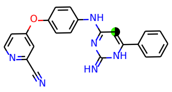
Medium | 14.06

(S37.22) **597**

# Pyrimidin-2(1H)-ones

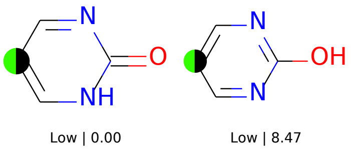


(S38.1) **99**

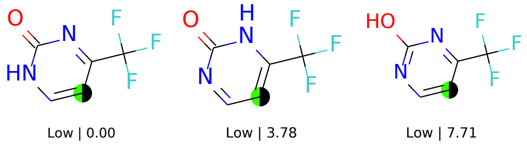


(S38.2) **100**

# Pyrimidin-4(3H)-ones

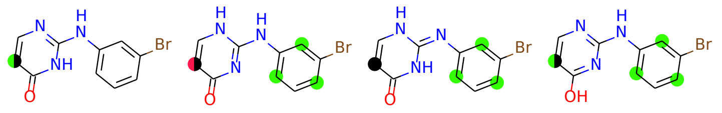

Medium | 0.00 Medium | 3.05 Medium | 5.80 Medium | 6.70

(S39.1) **101**

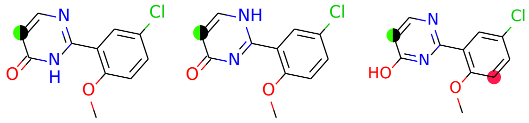

Medium | 0.00 Medium | 3.54 Medium | 9.29

(S39.2) **102**

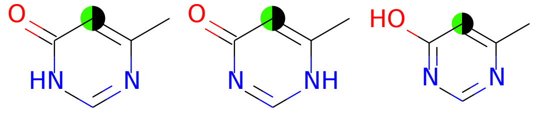

Medium | 0.00 Medium | 2.77 Medium | 8.46

(S39.3) **103**

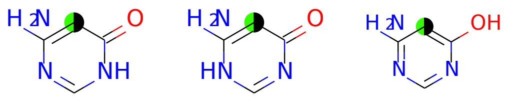

Medium | 0.00 Medium | 5.45 Medium | 8.93

(S39.4) **104**

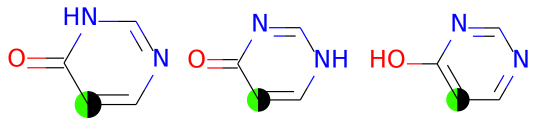

Low | 0.00 Medium | 2.93 Low | 8.26

(S39.5) **105**

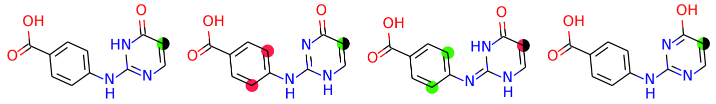

Medium | 0.00 Medium | 3.03 Medium | 5.29 Medium | 6.01

(S39.6) **106**

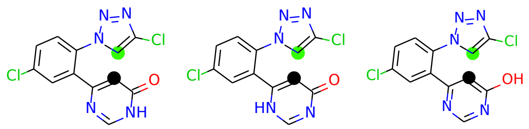

Medium | 0.00 Medium | 6.01 Medium | 8.50

(S39.7) **594**

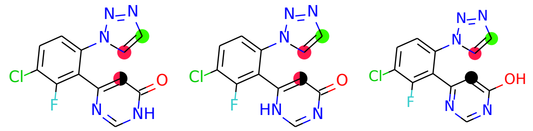

Medium | 0.00 Medium | 5.42 Medium | 7.98

(S39.8) **595**

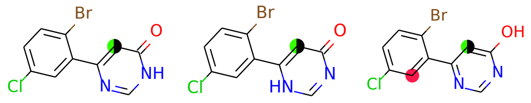

Medium | 0.00 Medium | 3.88 Medium | 8.17

(S39.9) **596**

# Pyrazines

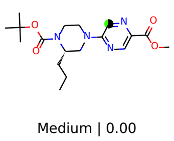


(S40.1) **30**

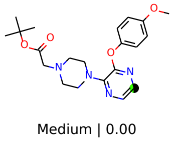


(S40.2) **31**

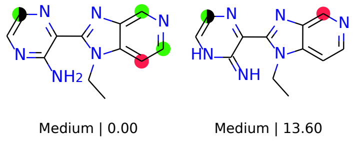


(S40.3) **49**

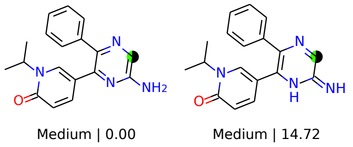


(S40.4) **107**

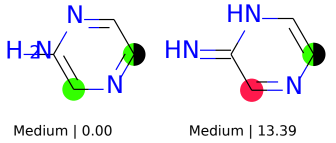


(S40.5) **108**

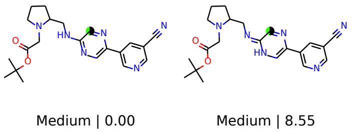


(S40.6) **109**

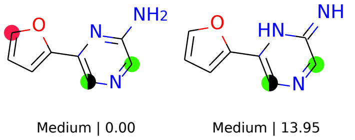


(S40.7) **131**

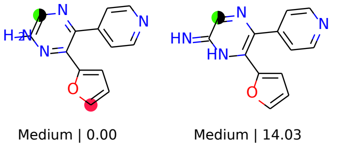


(S40.8) **132**

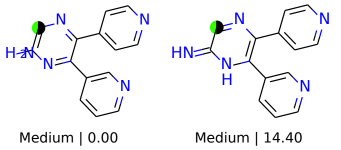


(S40.9) **133**

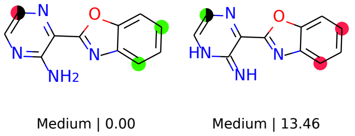


(S40.10) **134**

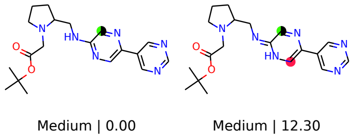


(S40.11) **135**

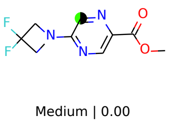


(S40.12) **192**

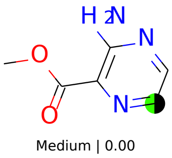


(S40.13) **193**

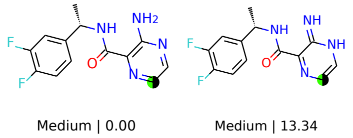


(S40.14) **194**

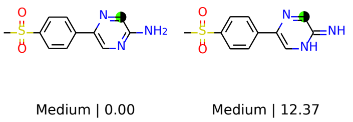


(S40.15) **196**

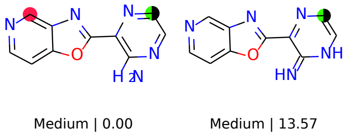


(S40.16) **198**

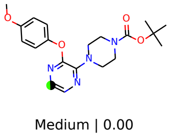


(S40.17) **199**

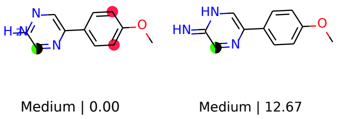


(S40.18) **200**

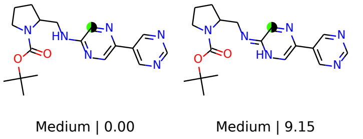


(S40.19) **201**

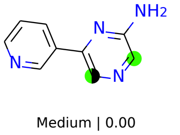


(S40.20) **202**

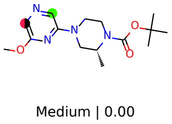


(S40.21) **203**

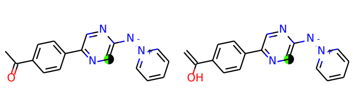

Medium | 0.00 Medium | 9.06

(S40.22) **205**

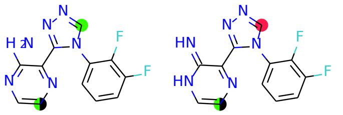

Medium | 0.00 Medium | 12.60

(S40.23) **206**

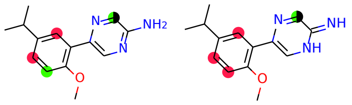

Medium | 0.00 Medium | 12.72

(S40.24) **207**

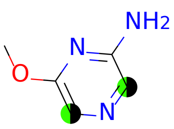

Medium | 0.00

(S40.25) **211**

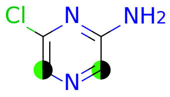

Medium | 0.00

(S40.26) **212**

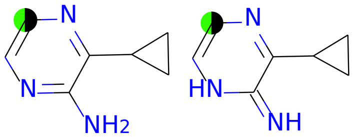

Medium | 0.00 Medium | 9.92

(S40.27) **284**

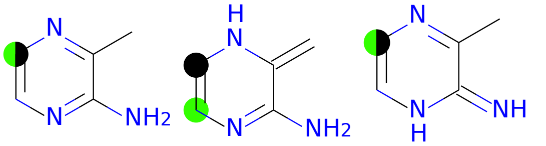

Medium | 0.00 Medium | 9.57 Medium | 12.68

(S40.28) **285**

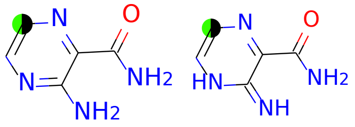

Medium | 0.00 Medium | 13.84

(S40.29) **286**

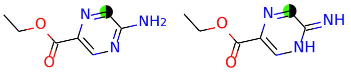

Medium | 0.00 Medium | 11.73

(S40.30) **289**

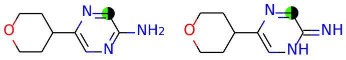

Medium | 0.00 Medium | 12.90

(S40.31) **290**

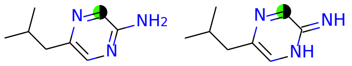

Medium | 0.00 Medium | 13.33

(S40.32) **291**

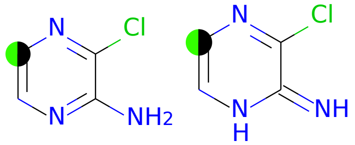

Medium | 0.00 Medium | 12.65

(S40.33) **292**

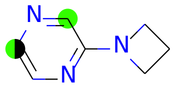

Medium | 0.00

(S40.34) **293**

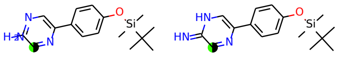

Medium | 0.00 Medium | 12.72

(S40.35) **195**

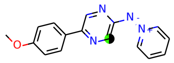

Medium | 0.00

(S40.36) **209**

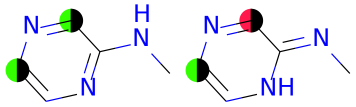

Medium | 0.00 Medium | 10.41

(S40.37) **283**

# Pyrazinones

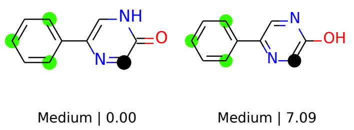


(S41.1) **190**

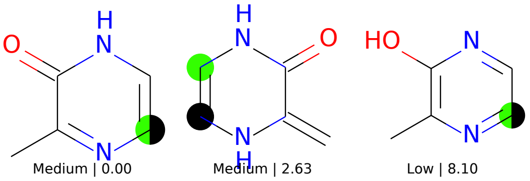


(S41.2) **197**

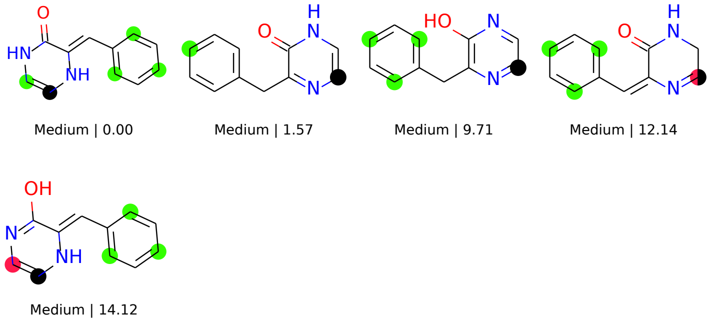


(S41.3) **204**

# 4H-Furo[3,2-b]pyrroles

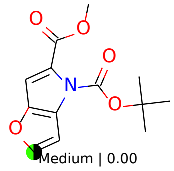


(S42.1) **472**

# 4H-Thieno[3,2-b]pyrroles

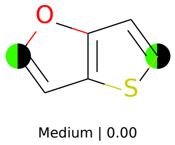


(S43.1) **471**

# Imidazo[2,1-b][1,3,4]thiadiazoles

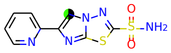

Medium | 0.00

(S44.1) **418**

# Indoles

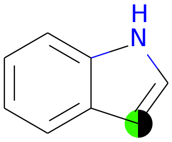

Medium | 0.00

(S45.1) **35**

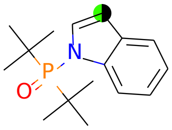

Medium | 0.00

(S45.2) **399**

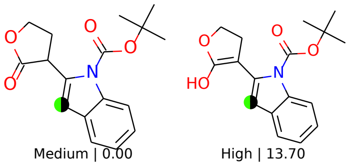

Medium | 0.00 High | 13.70

(S45.3) **407**

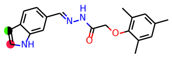

Medium | 0.00

(S45.4) **412**

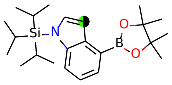

Medium | 0.00

(S45.5) **425**

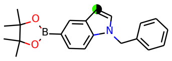

Medium | 0.00

(S45.6) **426**

# Benzofurans

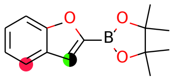

Medium | 0.00

(S46.1) **424**

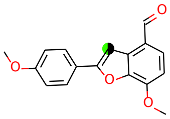

Medium | 0.00

(S46.2) **430**

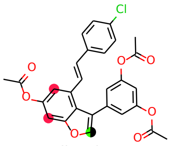

Medium | 0.00

(S46.3) **431**

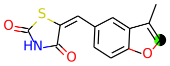

Medium | 0.00

(S46.4) **461**

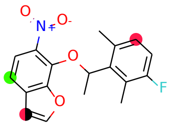

Medium | 0.00

(S46.5) **468**

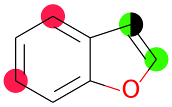

Medium | 0.00

(S46.6) **470**

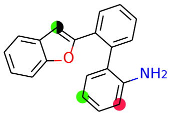

Medium | 0.00

(S46.7) **433**

# Benzo[b]thiophenes

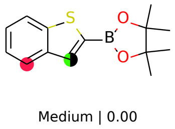


(S47.1) **423**

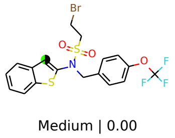


(S47.2) **429**

# Indazoles

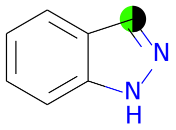
Medium | 0.00

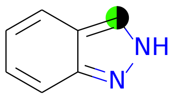
Medium | 3.49

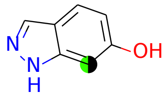
Medium | 0.00

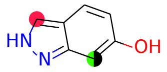
Medium | 3.31

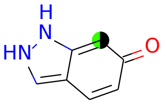
High | 13.74

(S48.1) **353**

(S48.2) **354**

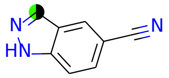
Medium | 0.00

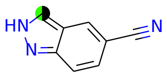
Medium | 3.33

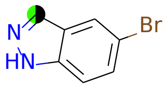
Medium | 0.00

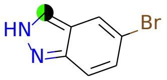
Medium | 3.31

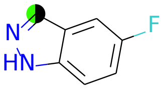
Medium | 0.00

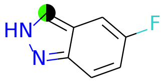
Medium | 3.12

(S48.3) **355**

(S48.4) **356**

(S48.5) **357**

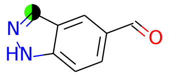
Medium | 0.00

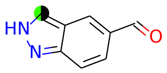
Medium | 3.15

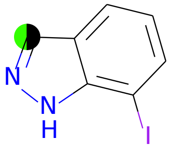
Medium | 0.00

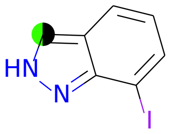
Medium | 2.92

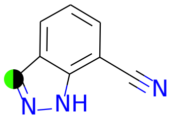
Medium | 0.00

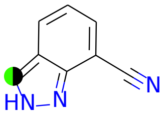
Medium | 1.48

(S48.6) **358**

(S48.7) **359**

(S48.8) **360**

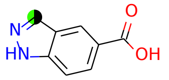
Medium | 0.00

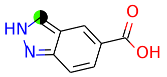
Medium | 3.39

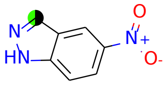
Medium | 0.00

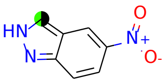
Medium | 3.06

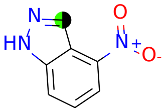
Medium | 0.00

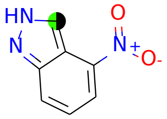
Medium | 2.64

(S48.9) **361**

(S48.10) **362**

(S48.11) **363**

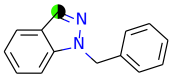
Medium | 0.00

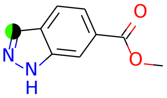
Medium | 0.00

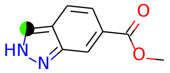
Medium | 2.70

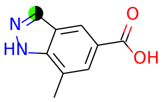
Medium | 0.00

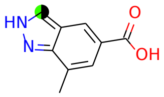
Medium | 2.82

(S48.12) **364**

(S48.13) **365**

(S48.14) **366**

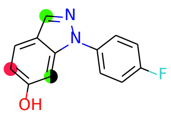
Medium | 0.00

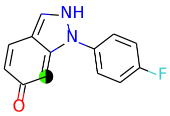
High | 14.87

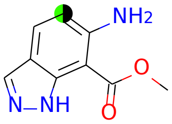
Medium | 0.00

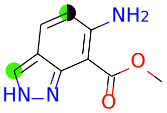
Medium | 3.55

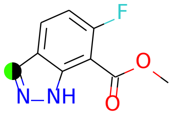
Medium | 0.00

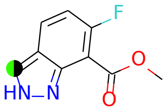
Medium | 3.69

(S48.15) **370**

(S48.16) **371**

(S48.17) **372**

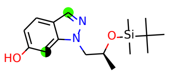
Medium | 0.00

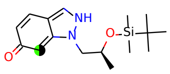
High | 14.00

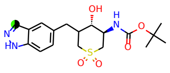
Medium | 0.00

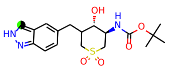
Medium | 4.13

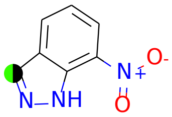
Medium | 0.00

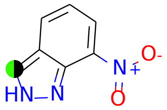
Medium | 2.87

(S48.18) **374**

(S48.19) **375**

(S48.20) **383**

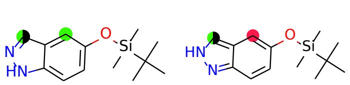

Medium | 0.00 Medium | 3.22

(S48.21) **367**

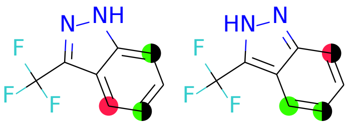

Medium | 0.00 Medium | 6.46

(S48.22) **368**

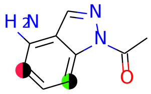

Medium | 0.00

(S48.23) **369**

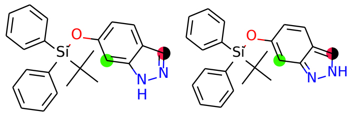

Medium | 0.00 Medium | 3.36

(S48.24) **373**

# 1H-Benzo[d]imidazoles

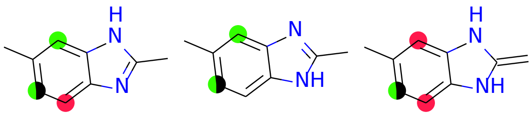

Medium | 0.00 Medium | 0.07 Medium | 7.61

(S49.1) **377**

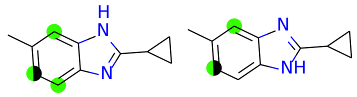

Medium | 0.00 Medium | 0.85

(S49.2) **378**

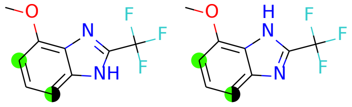

Medium | 0.00 Medium | 1.24

(S49.3) **381**

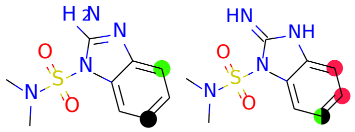

Medium | 0.00 Medium | 4.52

(S49.4) **382**

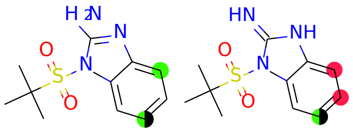

Medium | 0.00 Medium | 2.51

(S49.5) **385**

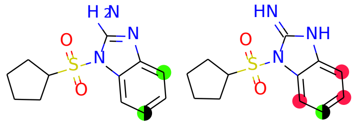

Medium | 0.00 Medium | 1.70

(S49.6) **387**

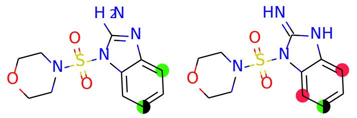

Medium | 0.00 Medium | 4.91

(S49.7) **388**

# 7-Azaindoles

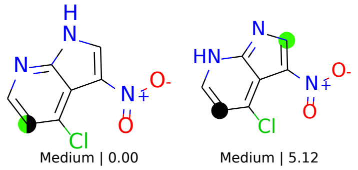


(S50.1) **334**

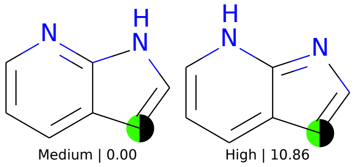


(S50.2) **335**

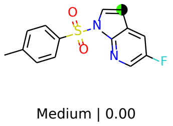


(S50.3) **336**

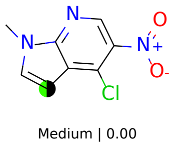


(S50.4) **337**

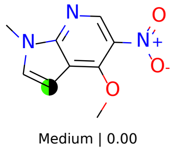


(S50.5) **338**

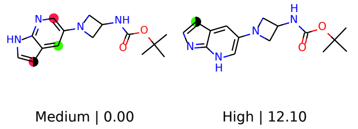


(S50.6) **339**

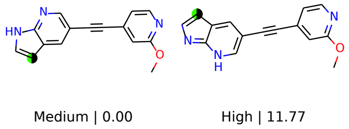


(S50.7) **340**

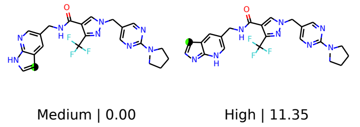


(S50.8) **341**

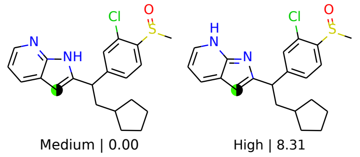


(S50.9) **342**

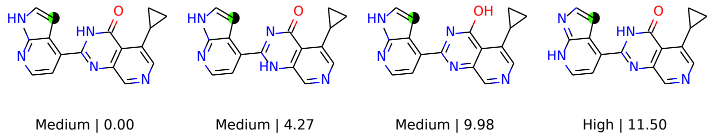


(S50.10) **343**

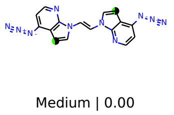


(S50.11) **344**

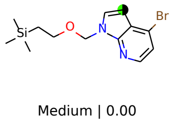


(S50.12) **345**

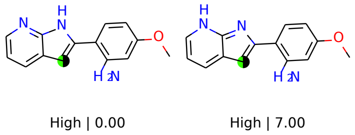


(S50.13) **346**

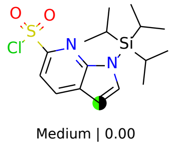


(S50.14) **347**

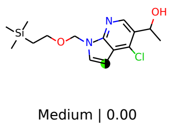


(S50.15) **348**

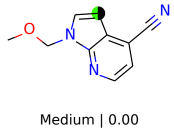


(S50.16) **349**

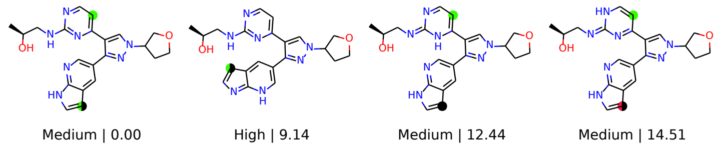


(S50.17) **351**

# 6-Azaindoles

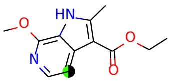

Medium | 0.00 Medium | 8.66

(S51.1) **316**

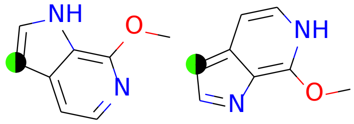

Medium | 0.00 High | 11.40

(S51.2) **322**

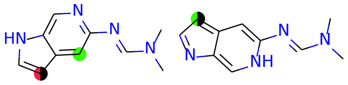

Medium | 0.00 High | 10.56

(S51.3) **323**

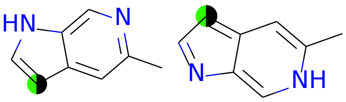

Medium | 0.00 High | 10.15

(S51.4) **325**

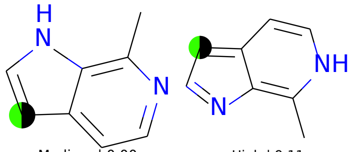

Medium | 0.00 High | 9.11

(S51.5) **326**

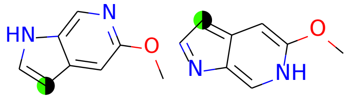

Medium | 0.00 High | 14.28

(S51.6) **327**

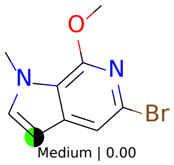

Medium | 0.00

(S51.7) **328**

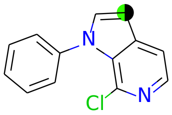

Medium | 0.00

(S51.8) **329**

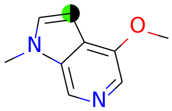

Medium | 0.00

(S51.9) **330**

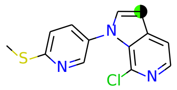

Medium | 0.00

(S51.10) **331**

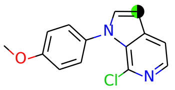

Medium | 0.00

(S51.11) **332**

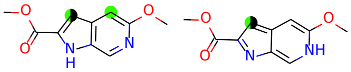

Medium | 0.00 High | 12.37

(S51.12) **333**

# 5-Azaindoles

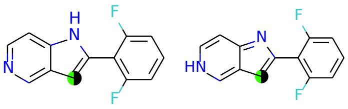

Medium | 0.00 High | 12.47

(S52.1) **317**

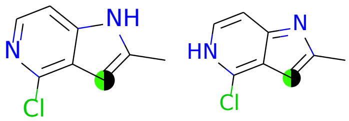

Medium | 0.00 High | 13.86

(S52.2) **318**

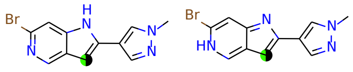

High | 0.00 High | 12.40

(S52.3) **319**

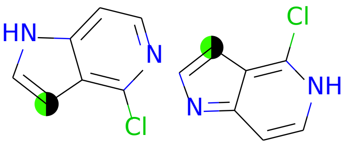

Medium | 0.00 High | 13.60

(S52.4) **320**

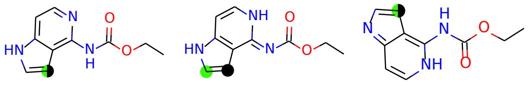

Medium | 0.00 Medium | 1.76 High | 11.41

(S52.5) **321**

# 4-Azaindoles

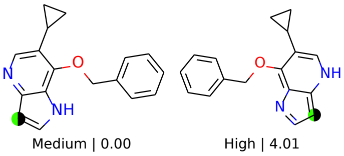


(S53.1) **300**

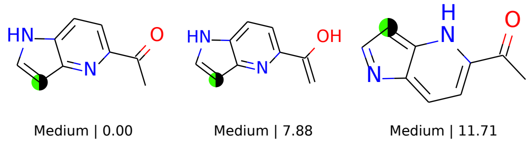


(S53.2) **301**

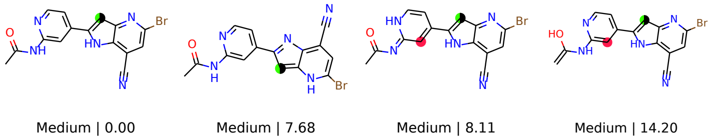


(S53.3) **302**

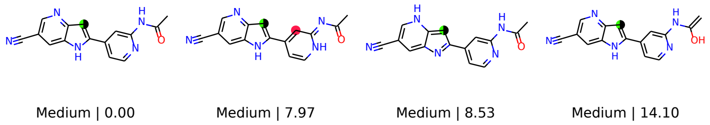


(S53.4) **303**

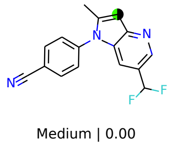


(S53.5) **304**

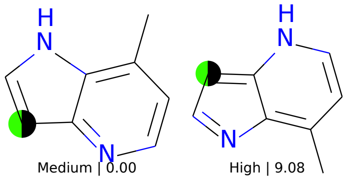


(S53.6) **305**

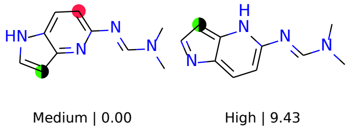


(S53.7) **306**

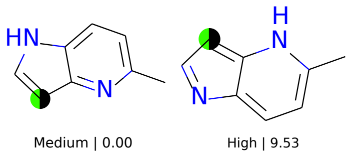


(S53.8) **307**

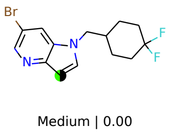


(S53.9) **308**

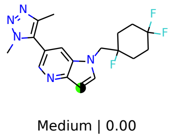


(S53.10) **309**

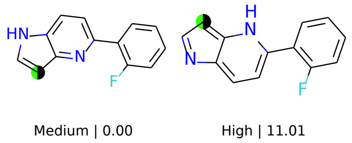


(S53.11) **310**

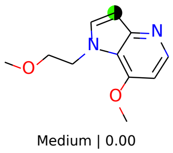


(S53.12) **311**

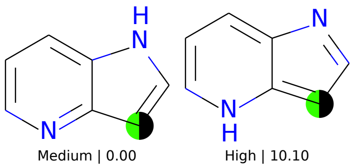


(S53.13) **312**

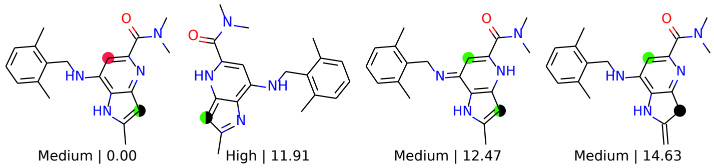


(S53.14) **313**

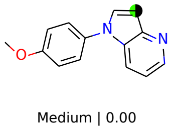


(S53.15) **314**

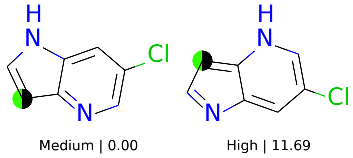


(S53.16) **315**

# Imidazo[1,2-a]pyridines

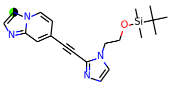

High | 0.00

(S54.1) **170**

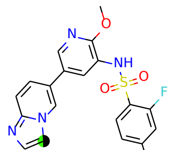

Medium | 0.00

(S54.2) **172**

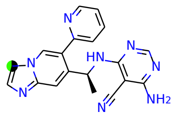

Medium | 0.00

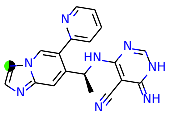

Medium | 11.13

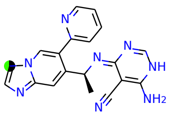

Medium | 11.37

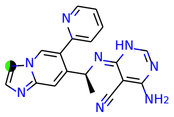

High | 11.45

(S54.3) **174**

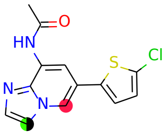

Medium | 0.00

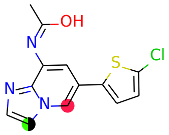

Medium | 12.12

(S54.4) **176**

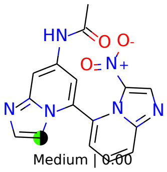

Medium | 0.00

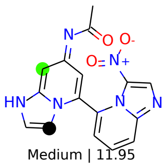

Medium | 11.95

(S54.5) **177**

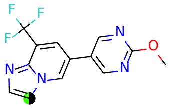

Medium | 0.00

(S54.6) **178**

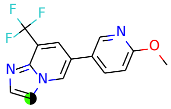

Medium | 0.00

(S54.7) **179**

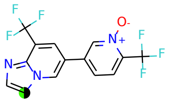

Medium | 0.00

(S54.8) **180**

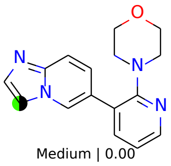

Medium | 0.00

(S54.9) **182**

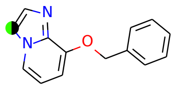

Medium | 0.00

(S54.10) **184**

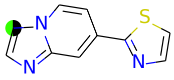

Medium | 0.00

(S54.11) **187**

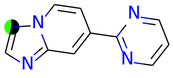

Medium | 0.00

(S54.12) **188**

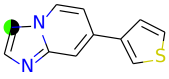

High | 0.00

(S54.13) **189**

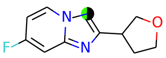

Medium | 0.00

(S54.14) **408**

# Furo[2,3-b]pyridines

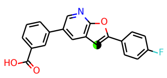

Medium | 0.00

(S55.1) **455**

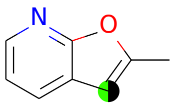

Medium | 0.00

(S55.2) **462**

# Furo[2,3-c]pyridines

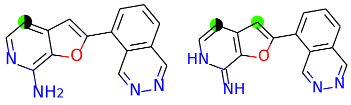
Medium | 0.00 Medium | 10.08

(S56.1) **268**

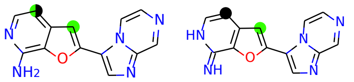
Medium | 0.00 Medium | 10.10

(S56.2) **269**

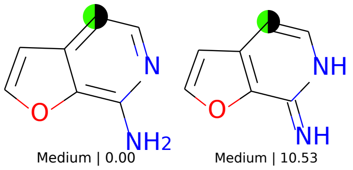
Medium | 0.00 Medium | 10.53

(S56.3) **270**

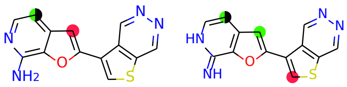
Medium | 0.00 Medium | 9.42

(S56.4) **271**

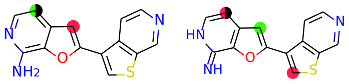
Medium | 0.00 Medium | 9.81

(S56.5) **272**

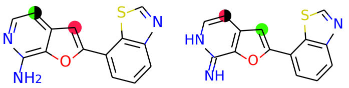
Medium | 0.00 Medium | 9.13

(S56.6) **273**

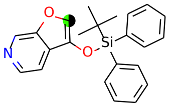
Medium | 0.00

(S56.7) **473**

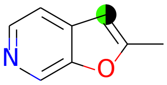
Medium | 0.00

(S56.8) **480**

# Furo[3,2-c]pyridines

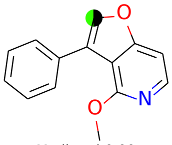
Medium | 0.00

(S57.1) **459**

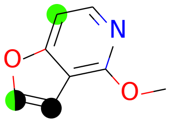
Medium | 0.00

(S57.2) **460**

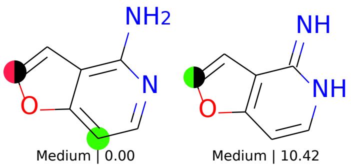
Medium | 0.00 Medium | 10.42

(S57.3) **482**

# Furo[3,2-b]pyridines

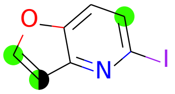
Medium | 0.00

(S58.1) **439**

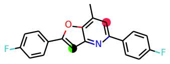
Medium | 0.00

(S58.2) **440**

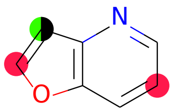
Medium | 0.00

(S58.3) **447**

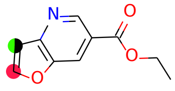
Low | 0.00

(S58.4) **448**

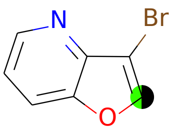
Medium | 0.00

(S58.5) **481**

# [1,2,4]Triazolo[4,3-a]pyridines

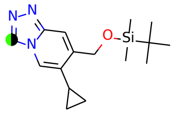

Medium | 0.00

(S59.1) **143**

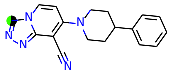

Medium | 0.00

(S59.2) **150**

# 1H-Pyrazolo[3,4-b]pyridines

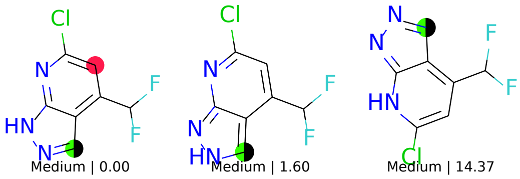

Medium | 0.00 Medium | 1.60 Medium | 14.37

(S60.1) **394**

# 1H-Imidazo[4,5-c]pyridines

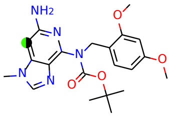

Medium | 0.00

(S61.1) **265**

# Imidazo[1,2-a]pyrazines

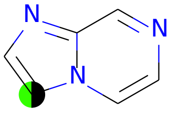

Medium | 0.00

(S62.1) **38**

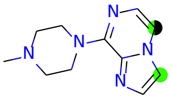

Medium | 0.00

(S62.2) **39**

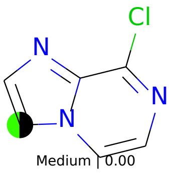

Medium | 0.00

(S62.3) **40**

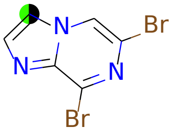

Medium | 0.00

(S62.4) **111**

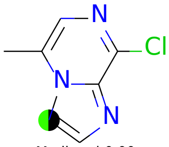

Medium | 0.00

(S62.5) **112**

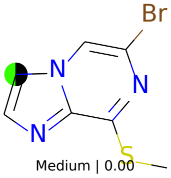

Medium | 0.00

(S62.6) **186**

# Imidazo[1,2-c]pyrimidines

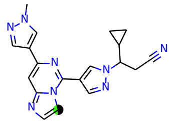

High | 0.00

(S63.1) **175**

# Imidazo[1,2-b]pyridazines

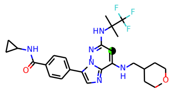

High | 0.00

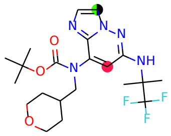

Medium | 0.00

(S64.1) **280** (S64.2) **281**

# 7H-Pyrrolo[2,3-d]pyrimidines

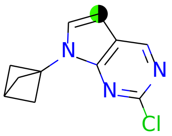

Medium | 0.00

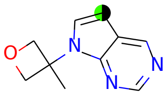

Medium | 0.00

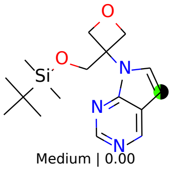

Medium | 0.00

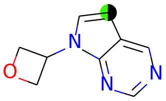

Medium | 0.00

(S65.1) **275** (S65.2) **403** (S65.3) **404** (S65.4) **405**

# Pyrazolo[1,5-c]pyrimidines

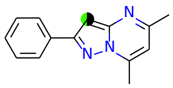

Medium | 0.00

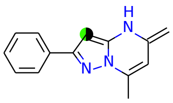

High | 7.42

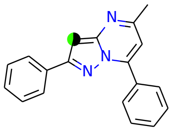

Medium | 0.00

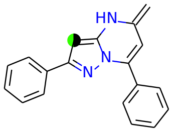

Medium | 7.14

(S66.1) **36** (S66.2) **115**

# Imidazo[1,5-a]pyrazines

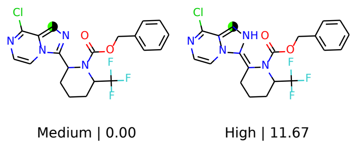


(S67.1) **279**

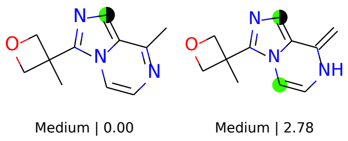


(S67.2) **406**

# Pyrrolo[2,1-f][1,2,4]triazines

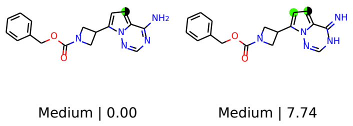


(S68.1) **411**

# Oxazolo[4,5-b]pyridines

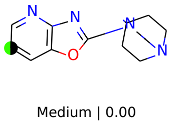


(S69.1) **264**

# Furo[2,3-d]pyrimidines

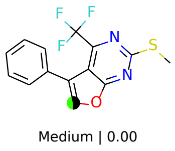


(S70.1) **457**

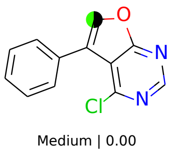


(S70.2) **463**

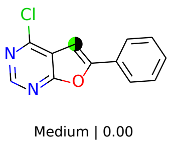


(S70.3) **464**

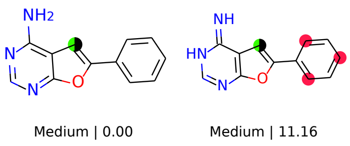


(S70.4) **467**

# Furo[2,3-b]pyrazines

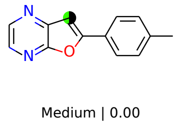


(S71.1) **487**

# [1,2,4]Triazolo[4,3-b]pyridazines

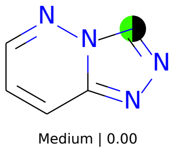


(S72.1) **41**

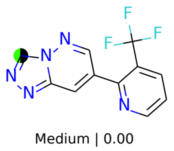


(S72.2) **116**

# Pyrazolo[1,5-a][1,3,5]triazines

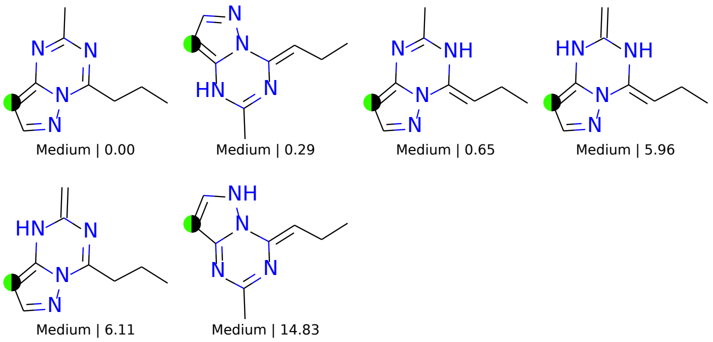


(S73.1) **113**

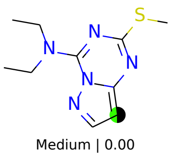


(S73.2) **114**

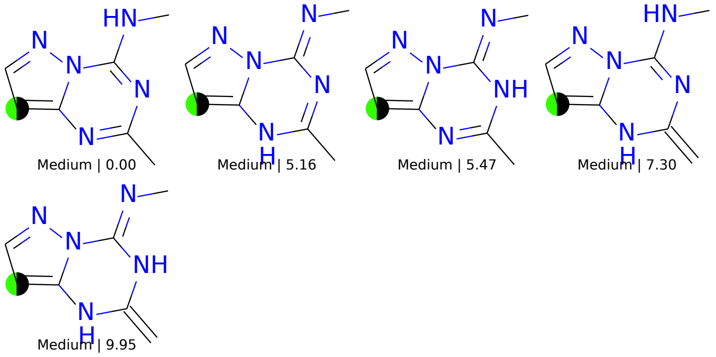


(S73.3) **117**

# Naphthalenes

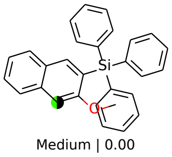


(S74.1) **510**

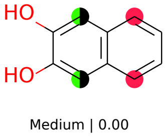


(S74.2) **512**

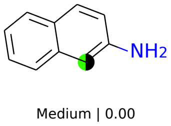


(S74.3) **538**

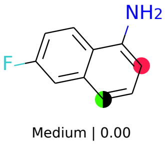


(S74.4) **544**

# Quinolines

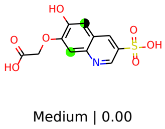


(S75.1) **415**

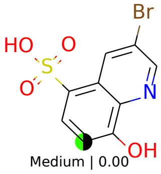


(S75.2) **416**

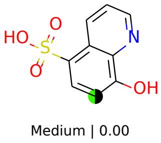


(S75.3) **417**

# Isoquinolines

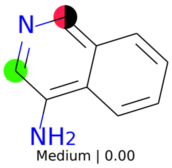


(S76.1) **266**

# Quinoxalines

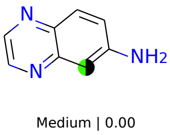


(S77.1) **548**

# 1,5-Naphthyridines

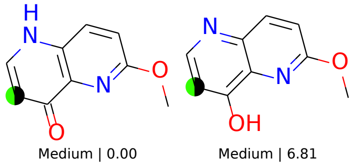


(S78.1) **260**

# Pyrido[4,3-d]pyrimidines

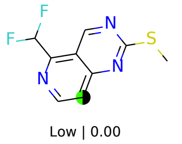


(S79.1) **396**

# Pyrido[3,4-b]pyrazines

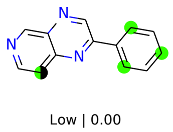


(S80.1) **262**

# Furo[3,2-c]pyridin-4(5H)-ones

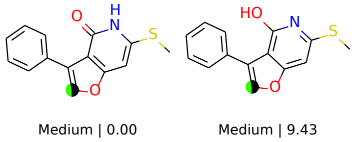


(S81.1) **456**

# Furo[2,3-d]pyrimidin-4(3H)-ones

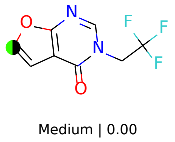


(S82.1) **445**

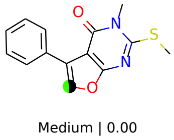


(S82.2) **458**

# Imidazo[1,2-a]pyrazin-8(7H)-ones

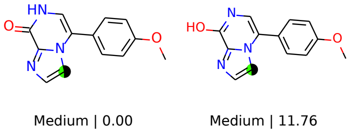


(S83.1) **181**

# Thiazolo[5,4-b]pyridin-5(4H)-ones

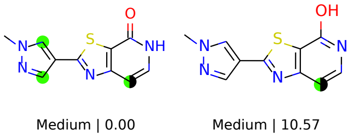


(S84.1) **585**

# 3,4-Dihydro-5H-[1,2,3]triazolo[4,5-b]pyridin-5-ones

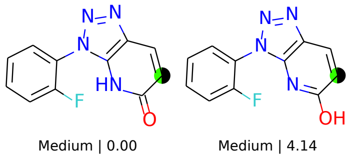


(S85.1) **267**

# 1,3-Dihydro-2H-benzo[d]imidazol-2-ones

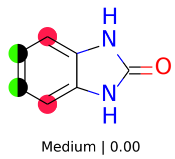


(S86.1) **376**

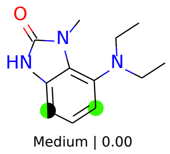


(S86.2) **386**

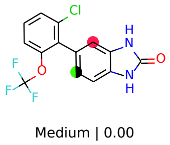


(S86.3) **389**

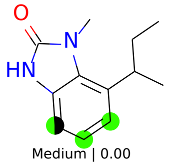


(S86.4) **384**

# Oxazolo[4,5-b]pyridin-2(3H)-ones

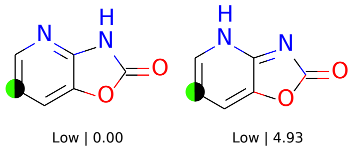


(S87.1) **261**

# Quinazolin-4(3H)-ones

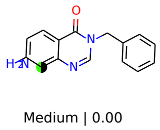


(S88.1) **580**

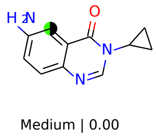


(S88.2) **581**

# 1,7-Naphthyridin-8(7H)-ones

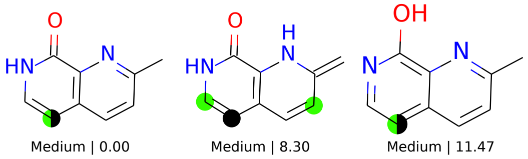


(S89.1) **263**

# Pyrido[2,3-d]pyrimidin-7(8H)-ones

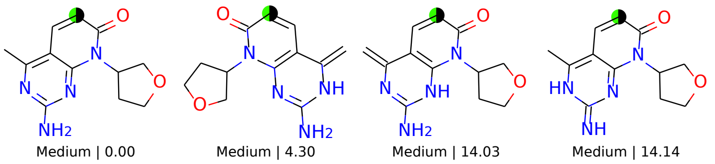


(S90.1) **409**

# 2H-Chromen-2-ones

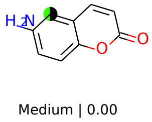


(S91.1) **557**

## Graphical TOC Entry
